# Clinically Relevant Humanized Mouse Models of Metastatic Prostate Cancer Facilitate Therapeutic Evaluation

**DOI:** 10.1158/1541-7786.MCR-23-0904

**Published:** 2024-05-31

**Authors:** Raymond J. Kostlan, John T. Phoenix, Audris Budreika, Marina G. Ferrari, Neetika Khurana, Jae E. Choi, Kristin Juckette, Somnath Mahapatra, Brooke L. McCollum, Russell Moskal, Rahul Mannan, Yuanyuan Qiao, Donald J. Vander Griend, Arul M. Chinnaiyan, Steven Kregel

**Affiliations:** 1 Department of Cancer Biology, Loyola University Chicago, Maywood, Illinois.; 2 Integrated Program in Biomedical Science, Biochemistry, Molecular and Cancer Biology, Loyola University Chicago, Maywood, Illinois.; 3 Michigan Center for Translational Pathology, University of Michigan, Ann Arbor, Michigan.; 4 Department of Pathology, University of Michigan, Ann Arbor, Michigan.; 5 Rogel Cancer Center, University of Michigan, Ann Arbor, Michigan.; 6 Department of Pathology, University of Illinois at Chicago, Chicago, Illinois.

## Abstract

There is tremendous need for improved prostate cancer models. Anatomically and developmentally, the mouse prostate differs from the human prostate and does not form tumors spontaneously. Genetically engineered mouse models lack the heterogeneity of human cancer and rarely establish metastatic growth. Human xenografts are an alternative but must rely on an immunocompromised host. Therefore, we generated prostate cancer murine xenograft models with an intact human immune system (huNOG and huNOG-EXL mice) to test whether humanizing tumor-immune interactions would improve modeling of metastatic prostate cancer and the impact of androgen receptor-targeted and immunotherapies. These mice maintain multiple human immune cell lineages, including functional human T-cells and myeloid cells.

**Implications:**

To the best of our knowledge, results illustrate the first model of human prostate cancer that has an intact human immune system, metastasizes to clinically relevant locations, responds appropriately to standard-of-care hormonal therapies, and can model both an immunosuppressive and checkpoint-inhibition responsive immune microenvironment.

Watch the interview with Steven Kregel, PhD, recipient of the 2026 *Molecular Cancer Research* Award for Outstanding Journal Article: https://vimeo.com/1208910912

## Graphical Abstract

**Figure:**
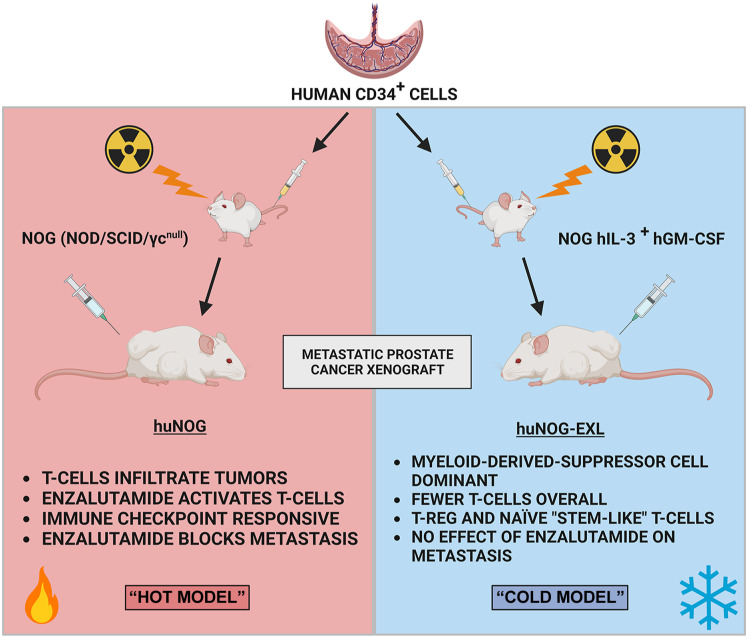

## Introduction

Prostate cancer is one of the leading causes of cancer-related deaths, and treatment options for men with advanced, metastatic disease are limited. Progression to metastatic-castration-resistant prostate cancer (mCRPC) is often driven by the maintenance of androgen receptor (AR) signaling, despite attempted blockades with standard-of-care treatments such as castration and enzalutamide (enza; refs. 1, 2). Development of novel therapeutics for mCRPC depends on improving the murine models of this disease. The current genetically engineered model (GEM) systems for mCRPC exhibit several shortcomings that hinder their clinical applicability. GEM systems rely on the murine prostate, which differs anatomically and developmentally from the human prostate, and does not form sporadic tumors (3, 4). GEMs lack the heterogeneity of human disease and rarely establish metastatic growth. Furthermore, disease progression in GEM systems tend to be driven in a contrived manner, unrelated to human disease or the commonly observed drivers of disease progression (5–7). While human xenografts represent alternative models, they rely on tumor growth in an immunocompromised murine host and are thus unsuitable for investigations into tumor-immune interactions and immunotherapy interventions, which has been a rapidly expanding area of cancer research (5).

Consequently, the development of a human-derived model that can recapitulate the natural history of the disease—from initiation to metastatic spread—and will respond appropriately to the standard-of-care hormonal therapies is required to accelerate translational progress in prostate cancer research (5–7). To address this issue, we employed a series of prostate cancer xenograft models in recently developed murine lineages with an intact human immune system (huNOG and huNOG-EXL mice, Taconic Biosciences, Germantown, NY). Male huNOG mice are produced by engrafting juvenile immunocompromised NOG (NOG-NOD/SCID/γnull/c) mice with human CD34^+^ hematopoietic stem cells (HSC) from human umbilical cord blood. These mice stably develop and maintain multiple human cell lineages, including functional human T-cells, and can be human leukocyte antigen (HLA) matched for a variety of xenograft models (8). Additional mouse models produced with human cytokine Interleukin-3 (IL3) and Granulocyte-macrophage colony-stimulating factor (hGM-CSF) huNOG-EXL provide more support to human myeloid cells, which are often outcompeted by the host in standard huNOG mice (8, 9). In the context of enza treatment, we revealed differential levels of metastatic outgrowth in NOG immunocompromised controls compared with newly developed huNOG mice. These results led us to hypothesize that the anti-metastatic responses of AR-targeted therapies are achieved through the human immune system and prompted us to profile the differences in immune microenvironment populations across our models. Here we describe new tools to assay the therapeutic responses of metastatic prostate cancer xenografts in huNOG and huNOG-EXL, which model an activatable T-cell and an immunosuppressed subcutaneous tumor microenvironment, respectively.

## Materials and Methods

### Cell lines and culture

R1881 was purchased from Sigma-Aldrich, and enzalutamide (MDV3100) and anti-Programmed Death-1 [anti-PD1, Pembrolizumab (Pembro)] were purchased from Selleck Chemicals. They were stored at −20°C in ethanol, −80°C in DMSO, and constituted fresh from lyophilized powder in PBS, respectively. CWR-22Rv1 (22Rv1) and VCaP cell lines were purchased from American Type Culture Collection (Manassas, VA) and validated and cultured as described (2, 10). In addition, they were transduced with lentivirus for luciferase (luc2) as previously described (2). CWR-R1, VCaP, LAPC-4, LNCaP, and enzalutamide-resistant counterparts, in addition to BPH-1, 957E/hTERT, NCI-H660 (H660), PC3, DU145, PNT-2, and RWPE1 cells, were generously provided by Dr. Donald J. Vander Griend at the University of Illinois at Chicago and have been previously characterized and cultured as described (2, 11, 12). Dr. Peter Nelson at Fred Hutchinson Cancer Center provided LNCaP-sh and LNCaP-APIPC cells (13). All cultures were routinely screened for mycoplasma contamination using the ATCC Universal Mycoplasma Detection Kit.

### Murine prostate tumor xenograft models

NOG control, huNOG and huNOG-EXL humanized mice, were obtained from Taconic Biosciences. huNOG and huNOG-EXL came engrafted from two different independent HSC donors, and an *N* = 3 for each donor and control per condition to maintain minimal statistical power and illustrate reproducibility. Mice were anesthetized using 2% Isoflurane (inhalation), and either 1 × 10^6^ VCaP or 5 × 10^5^ 22Rv1 cells suspended in 100 μL of PBS with 50% Matrigel (BD Biosciences) were implanted subcutaneously into the dorsal flank on both sides of the mice. Once the tumors reached a palpable stage (100 mm^3^), the animals were randomized and treated with enzalutamide or vehicle control [1% Carboxymethylcellulose (Sigma-Aldrich), 0.25% TWEEN-80 (Sigma-Aldrich), and 98.75% PBS] by oral gavage. Growth in tumor volume was recorded using digital calipers, and tumor volumes were estimated using the formula (π/6) (*L* × *W*^2^), where *L* = length of tumor and *W* = width. Body weight during the study was also monitored. At the end of the studies, once the largest tumors met defined endpoints of 1 cm^3^ (22 days, post injection for 22Rv1 cells), mice were sacrificed, tissues imaged *ex vivo*, and tumors were extracted for the downstream analyses. For testosterone implantation, mice were surgically castrated and concurrently implanted with silastic tubing containing 25 mg testosterone (Steraloids Inc) for sustained release. 22Rv1 cell implantation occurred after 1–1.5 weeks of allowing circulating testosterone levels to equilibrate to approximate human hormone levels (14).

For the VCaP-CRPC experiment, VCaP tumor-bearing mice were castrated when the tumors were approximately 200 mm^3^ in size after 14 days. Once the tumor grew back to the pre-castration size, 7 days later, the animals were treated with either vehicle (oral gavage or PBS intraperitoneal injection), enzalutamide (10 mg/kg 5 days a week via oral gavage), pembrolizumab (1 mg/kg 3 times a week, intraperitoneal injection), or both, with respective controls. Mice were sacrificed at endpoint when tumors approached 1 cm^3^, which was 52 days following initial injection. All procedures involving mice were approved by the University Committee on Use and Care of Animals (UCUCA) at the University of Michigan or Loyola University Chicago and conform to all regulatory standards.

### *Ex vivo* imaging

At tumor endpoint, mice were injected with 150 mg/kg body mass D-luciferin (Promega) via intraperitoneal injection. Then, they were humanely sacrificed, mice necropsied, and tissues were rapidly imaged (within 10 minutes post-sacrifice) with the bioluminescence signal being assessed with the IVIS Spectrum In Vivo Imaging System (PerkinElmer). Tissue tumor burden was calculated based on the total flux [photons per second (p/s)] normalized to area (average radiance), utilizing Living Image software and statistics performed using Graph Pad Prism by utilizing analysis of variance (ANOVA) with multiple testing corrections across samples and Mann–Whitney and Kolmogorov–Smirnov *t*-tests between groups.

### Flow cytometry

#### huNOG experiments

After *ex vivo* imaging, mononuclear cells were isolated from the subcutaneous tumors and spleen and were stained with fluorescently conjugated antibodies as previously described (15). CountBright Absolute Counting Beads (Thermo Fisher Scientific) were used to quantify cell number. For cytokine staining, lymphocytes were incubated at 37°C for 4 hours in culture medium containing PMA (5 ng mL^−1^), ionomycin (500 ng mL^−1^), Brefeldin A (1:1,000), and Monensin (1:1,000). Extracellular staining was performed for 20 minutes using the antibodies listed below. Cells were then washed and resuspended in 1 mL of freshly prepared Fix/Perm solution (BD Biosciences) and incubated overnight at 4°C. The cells were then washed Perm/Wash buffer (BD Biosciences) and stained with intracellular antibodies listed below. Data collection and analysis was performed on a LSRII equipped with four lasers or a Fortessa equipped with four lasers (BD Bioscience) using BD FACS Diva software. The following human antibodies were used: CD45 (BD Biosciences), CD3 (Thermo Fisher Scientific), CD8 (Biosciences), CD4 (Thermo Fisher Scientific), and IFNγ (BD Biosciences). All antibodies were used at a 1:100 dilution, as previously described (16).

#### huNOG-EXL experiments

The huNOG-EXL experiments were similar to those above; however, we utilized the Cytek 5 Laser Aurora full-spectrum flow cytometer (Cytek Biosciences). Tumor and spleen samples were frozen in liquid nitrogen and later thawed and harvested to acquire mononuclear cells. After harvesting, cells were aliquoted and resuspended in Human TruStainFcX (Clone Information Proprietary, BioLegend) and TruStain FcXPlus (Clone S17011E, BioLegend) according to the provided recommendation (5 μLper 100 μL PBS per 1 million cells). Cells were then washed and stained in L/D Fixable Dead Cell Stain (Thermo Fisher Scientific) for 30 minutes. Next cells were washed, and extracellular staining was performed using a panel of antibodies listed below for 30 minutes. Data collection was performed using SpectroFlo 3.0.1. The following antibodies were used: mCD45 (Clone 30-F11, BD Biosciences) hCD45 (Clone HI30, BioLegend), CD3 (Clone UCHT1, BioLegend), CD4 (Clone RPA-T4, BioLegend), CD8a (Clone RPA-T8, BioLegend), CD11b (Clone TCRF44, BioLegend), CD14 (Clone 63D3, BioLegend), CD16 (Clone B13.1, BioLegend), CD19 (Clone HIB19, BioLegend), CD25 (Clone BC96, BioLegend), CD44 (Clone IM7, BioLegend), CD56 (Clone 5.1H11, BioLegend), CD69 (Clone FN50, BioLegend) PD-1, and (Clone NA105, BioLegend). Antibodies were titrated for optimal fluorescence per 1 million cells.

### Multiplex immunofluorescence

Multiplex immunofluorescence (multi-IF) was conducted on 4-μm-thick tissue sections that were whole formalin fixed and paraffin embedded (FFPE). Anti-CD3 rabbit monoclonal (Ventana, catalog no. 790-4341), anti-CD8 rabbit monoclonal (Ventana, catalog no. 790-4460), and anti-granzyme rabbit polyclonal antibodies (Cell Marque, catalog no. 262A-1b) were used. The multiplexing process was carried out consecutively in a sequence of granzyme, CD8, and CD3 with heat denaturation before the second primary antibody incubation. The process was automated using the Discovery Ultra automated slide staining system (Roche-Ventana Medical Systems), and OmniMap anti-rabbit and anti-mouse HRP kits (Ventana, catalog no. 760-4311 and 760-4310) were used. Discovery Cy5 Kit (RTU, Roche-Ventana Medical Systems, catalog no. 760-238) was used for granzyme, Discovery Red 610 (RTU, Roche-Ventana Medical Systems, catalog no. 760-245) for CD8, and Discovery FITC (RTU, Roche-Ventana Medical Systems, catalog no. 760-232) for CD3 were the fluorophores used. The staining was independently assessed by two study pathologists (RM and SM) at ×100 and ×200 magnification to check for the presence and pattern of expression.

### Immunohistochemistry and histology

Immunohistochemistry was performed on whole formalin-fixed, paraffin-embedded (FFPE) 4-μm-thick tissue sections using anti-AR rabbit monoclonal antibody (Ventana, catalog no. 790-4605) and anti-PD1 mouse monoclonal antibody (Ventana, catalog no. 760-7894). Heat-induced epitope retrieval was used, along with conditioning media (CC1) and signal development by OmniMap anti-rabbit (Ventana, catalog no. 760-4311) and anti-mouse HRP kits (Ventana, catalog no. 760-4310). The presence or absence of a brown signal in the nucleus and cytoplasm within the tumor was assessed by the study pathologists (RM and SM). Hematoxylin and eosin staining of tumors and metastases were performed as previously described (2).

### Western blotting

Whole-cell lysates collected from cells seeded at 1 × 10^6^ cells per well of a six well plate (Becton Dickinson), were lysed in RIPA-PIC buffer [150 mmol/L sodium chloride, 1.0% Igepal CA-630 (Sigma-Aldrich), 0.5% sodium deoxycholate, 0.1% SDS, 50 mmol/L Tris, pH 8.0, 1× protease inhibitor cocktail (Roche Molecular Biochemicals)], scraped, and sonicated (Fisher Scientific; model FB-120 Sonic Dismembrator). Protein was quantified by BCA assay (Thermo Fisher Scientific), and 50 μg of protein were loaded per lane. The following antibodies were used: anti-AR (D6F11 XP, Cell Signaling Technology); anti-Beta Actin (AC-15, Sigma-Aldrich); pan-anti-HLA -A-B-C (HLA class I ABC Polyclonal antibody, 15240-1-AP, Proteintech); and anti-PSA (*KLK3*; D11E1 XP, Cell Signaling Technology). Secondary antibodies and nitrocellulose membranes from LICOR were used and data captured using a LICOR Odyssey M system as previously described (12).

### Data availability

All primary data, including raw and unprocessed data and images, are available upon request.

## Results

Primary tumor and metastasis assays reveal 22Rv1-engrafted huNOG mice better reflect patient response to castration and enzalutamide treatment than NOG control mice.

**Figure 1.**
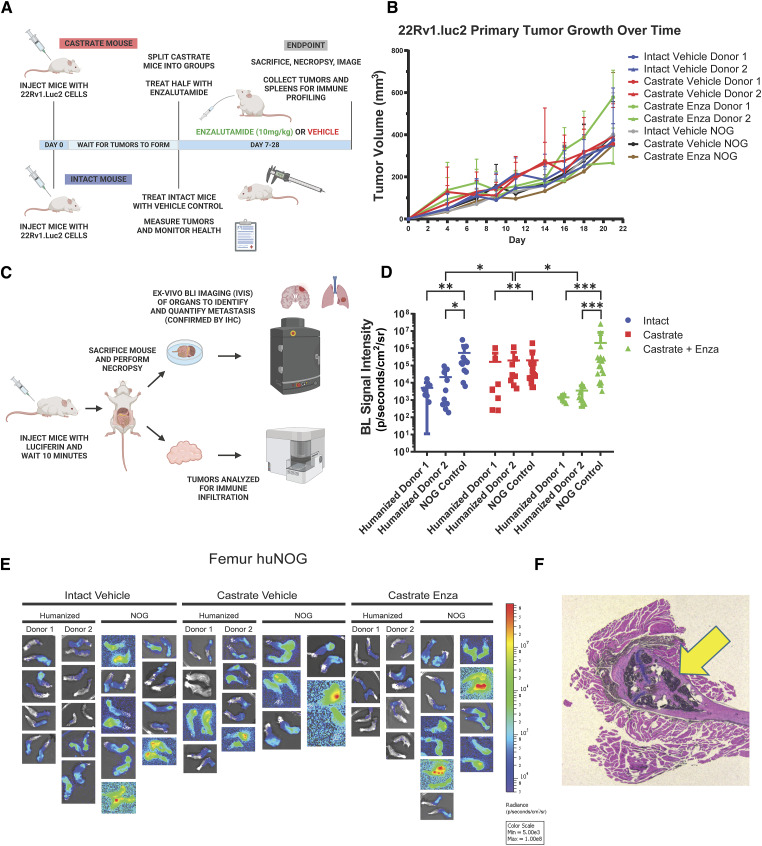
Experimental setup and 22Rv1 growth in huNOG mice. **A,** Experimental schematic: HuNOG and NOG control mice were surgically castrated. One week following castration, control intact and castrated mice were injected subcutaneously with luciferase-transduced 22Rv1 human prostate cancer cells to assay organ-specific metastatic growth. Castrated mice were then randomized and treated with enzalutamide or vehicle control (intact mice also treated with vehicle). Primary tumors were measured every 2–3 days until endpoint. huNOG: Intact *n* = 9, Castrated *n* = 8, Castrated Enza *n* = 7. NOG: Intact *n* = 9, Castrated *n* = 5, Castrated Enza *n* = 9. BioRender.com**B,** Subcutaneous primary flank tumor volume growth measured over time. huNOG: Intact *n* = 18, Castrated *n* = 16, Castrated Enza *n* = 14. NOG: Intact *n* = 18, Castrated *n* = 10, Castrated Enza *n* = 18. **C,** Schematic of endpoint analysis: Prior to sacrifice, mice were injected with luciferin. At sacrifice, organs were analyzed *ex vivo* for metastatic growth using the IVIS bioluminescence system (PerkinElmer); images illustrate signal intensity and location. BioRender.com**D,** Quantification of average signal intensity per unit area of bioluminescence of mouse femurs. **E,** Representative IVIS images of 22Rv1 metastasis to femur. **F,** Histological validation (H&E stain) of the femoral metastases confirmed by a pathologist (Rahul Manan), with cancer cells seen in both the bone marrow and matrix of the epiphyseal head of a mouse femur (yellow arrow indicates 22Rv1 tumor mass). (**A** and **C**, Created with BioRender.com.)

**Figure 2.**
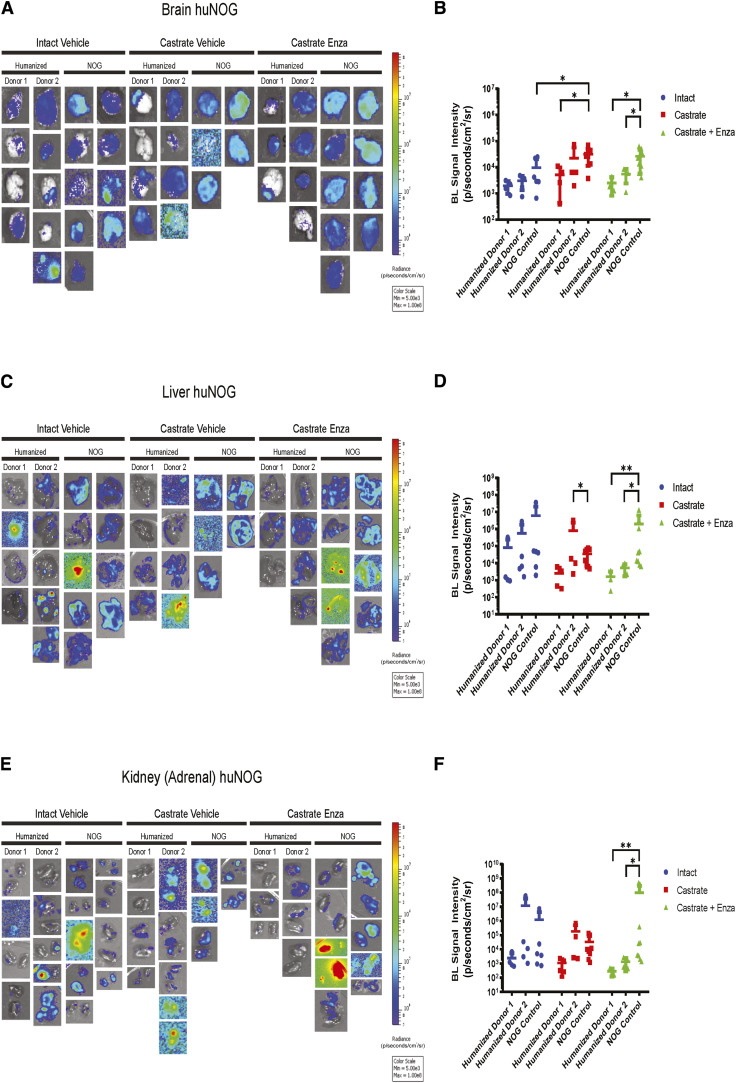
Metastasis by 22Rv1 to additional clinically relevant organs in huNOG mice. **A,** Bioluminescent images taken of mouse brain. **B,** Quantified bioluminescence of brain metastasis. **C,** Bioluminescent images taken of mouse liver. **D,** Quantified bioluminescence of liver metastasis. **E,** Kidney (adrenal gland) bioluminescent images. **F,** Quantified bioluminescence of Kidney (tumor mostly in adrenal gland).

We first sought to test the growth of the aggressive, bone metastatic CWR-22Rv1 (22Rv1) cell line (2) in huNOG mice compared with NOG [NOG-NOD/SCID/γnull/c (17)] controls with increasing levels of androgen-deprivation via surgical castration and surgical castration with enza treatment (10 mg/kg- MDV3100, Selleck Chemical). 22Rv1 are the most aggressive prostate cancer cell line *in vivo* that still maintains AR expression. They express wild-type full length AR, as well as H875Y mutant, and many splice variants including V7, some of which are stably through genetic alterations (18). 22Rv1 are considered enzalutamide resistant and respond weakly to both anti-androgens and androgens (19). This cell line was produced from a xenograft made from the primary tumor of a patient with extensive bone metastases upon disease presentation. In mice, these cells metastasize and colonize clinically relevant sites such as the bone, liver, lungs, and brain (2). We assayed “primary” tumor growth via subcutaneous flank injection (Fig. 1A and B) and performed metastasis detection and quantification (Figs. 1C–E and 2), with histological validation [representative image in Fig. 1F, higher resolution images of hematoxylin and eosin (H&E) stains of subcutaneous tumors, femur and liver metastasis in Supplementary Fig. S1, from luciferase-tagged 22Rv1.luc2 (22Rv1) cells transduced with Promega luciferase2 for bioluminescent imaging] to assay organ-specific metastatic growth (images of the femurs shown in Fig. 1E; see Fig. 2 for brain, liver, and kidneys, Supplementary Fig. S2 for humerus, Supplementary Fig. S3 for skull, Supplementary Fig. S4 for spleen, Supplementary Fig. S5 for lung, Supplementary Fig. S6 for heart), of huNOG (two different human male CD34^+^ HSC donors) and NOG control mice.

The subcutaneous “primary” tumor growth was affected neither by the castration nor enzalutamide treatment in NOG or huNOG mice at endpoint, but there was variability in the presence of an intact immune system in tumor size (Fig. 1B). At sacrifice, organs were *ex vivo* analyzed for metastatic growth using the IVIS bioluminescence system (schematic in Fig. 1C), and strikingly, metastatic growth was inhibited in huNOG mice under the conditions of castration in combination with enzalutamide treatment, (Figs. 1 and 2) mirroring clinical responses, which suggest metastatic outgrowth of castration-resistant prostate cancer can be attenuated by enza treatment (20). Surprisingly, AR antagonism with enza in immunocompromised NOG mice, contrary to clinical observations, yet observed in other models (21, 22), illustrated increased metastatic colonization and growth (Fig. 1D). Histology (H&E stain) confirmed femoral metastases (Fig. 1F), with cancer cells seen in both the bone marrow and matrix of the epiphyseal head of a mouse femur (Fig. 1F). The effect on growth was also observed for brain (Fig. 2A and B), liver (Fig. 2C and D), and kidney/adrenal (Fig. 2E and F) metastasis in the castration/enzalutamide group for the huNOG mice. These data suggest that 22Rv1 cells have the capacity to metastasize, with or without the presence of an intact human immune system, but the immune system may be activated to prevent outgrowth at secondary sites. These data also suggest that huNOG mice better recapitulate the patient castration/enzalutamide response and warrant further investigation into the immune populations that may be responsible for this phenotype.

### Profiling T cells in the tumors of huNOG mice revealed an activated immune profile

**Figure 3.**
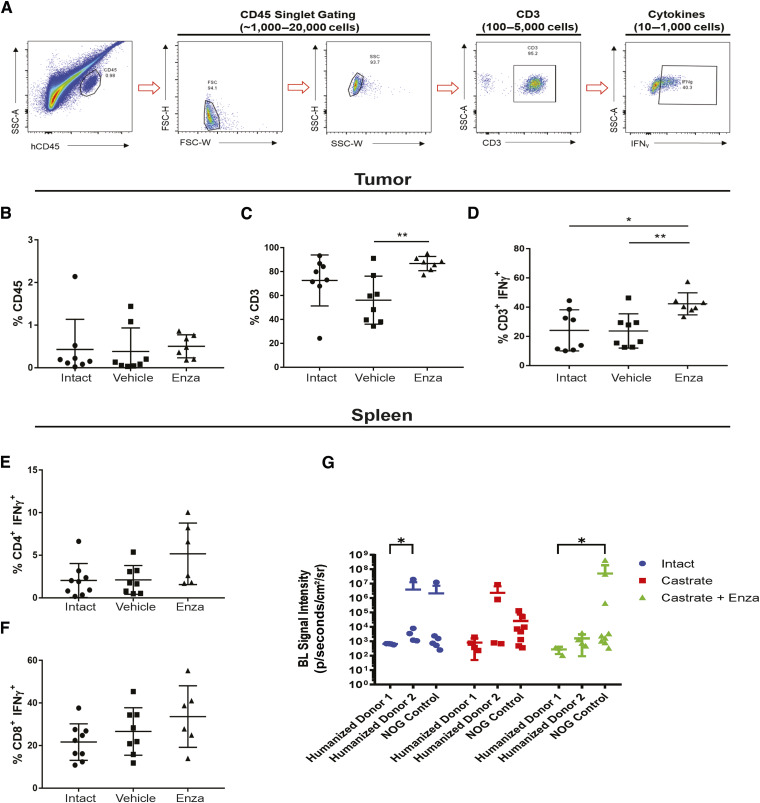
Activated T-cell immune profile in huNOG tumors. **A,** Gating strategy employed to determine T-cell activation status. **B,** Total percentage of CD45^+^ cells acquired from tumor tissue harvesting split between the intact control, castrated vehicle, and castrated with enzalutamide treatment. **C,** Percentage of CD3^+^ cells observed in the total CD45^+^ population. **D,** Percentage of CD3^+^ cells showing IFNγ expression via intracellular staining. **E,** Measurement of the percentage of CD4^+^ cells expressing IFNγ harvested from the spleen. **F,** Measurement of CD8^+^ cells expressing IFNγ from the spleen. **G,** Spleen bioluminescent metastasis signal quantification.

Because 22Rv1-engrafted huNOG mice exhibited a significant decrease in metastatic colonization compared with control NOG mice (Fig. 1B), we hypothesized that tumor-infiltrating lymphocytes (TIL) in huNOG mice may be responsible for this suppression, given their anti-tumor effects in patients (23, 24). To evaluate TILs in 22Rv1-engrafted huNOG mice, tumors and spleen were collected for immune profiling from treatment groups as described in Fig. 1A (either castrate or intact, with the castrate group further divided into enzalutamide treated or untreated). Disassociated tumors from the 22Rv1-engrafted huNOG mice were stained with human α-CD45 (for leukocytes) and α-CD3^+^ (T cells) were co-stained for intracellular IFNγ (marker of activation), and positively gated for FACS analysis (Fig. 3A). Analysis of the percentage of CD45^+^ leukocytes (Fig. 3B), CD3^+^ T-cells (Fig. 3C), and activated T cells (CD3^+^ IFNγ^+^; Fig. 3D) or (CD3^+^TNF-α^+^; Supplementary Fig. S7) showed that there were significant increases in the intra-tumoral CD3^+^ T cells and in their activation state (CD3^+^ IFNγ^+^, but not TNF-α^+^) in the mice treated with enzalutamide in the castrated group. Overall, the percentage of human CD45^+^ immune cells in the tumor was low (Fig. 3B), and T cells represented the vast majority of the immune cells in the tumor (Fig. 3C) in the huNOG model; thus, we focused our analysis solely on the CD3^+^ population. There were no significant differences in the number of splenic CD4^+^ helper T cells (Fig. 3E) and CD8^+^ cytotoxic T cells (Fig. 3F) for each of the different hormonal and enzalutamide treatment conditions, as a proxy for the whole-body influence of hormones in the mice. However, these data may also be affected by the presence of tumor cells in the spleen (Fig. 3G illustrates splenic metastases), as there were variable slight differences across donors and hormone conditions.

### Xenografts in huNOG-EXL mice reveal a suppressed immune profile

**Figure 4.**
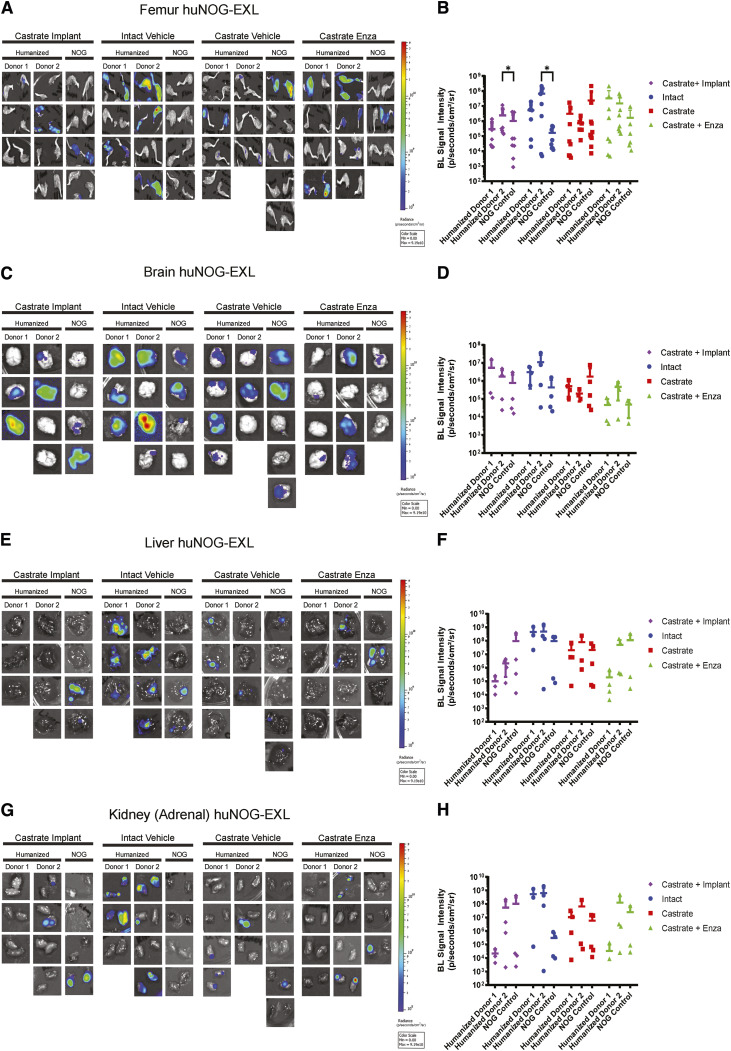
Metastasis by 22Rv1 to additional clinically relevant organs in huNOG-EXL mice. **A,** Bioluminescent images taken of femurs. **B,** Quantified bioluminescence of femur metastasis. **C,** Bioluminescent images taken of mouse brain. **D,** Quantified bioluminescence of brain metastasis. **E,** Bioluminescent images taken of mouse liver. **F,** Quantified bioluminescence of liver metastasis. **G,** Kidney (adrenal gland) bioluminescent images. **H,** Quantified bioluminescence of kidney (tumor mostly in adrenal gland).

Our results in 22Rv1-engrafted huNOG mice suggest that this mouse model with intact human lymphocytic cells models more closely the patient castration/enzalutamide response than a model using immunocompromised mice; however, immunotherapy responses in prostate cancer patients are low and are often characterized by dense myeloid infiltration (25). We evaluated tumor growth and metastasis in a humanized-NOG mouse system able to maintain not only cells of human lymphocyte lineage, but also human myeloid cells. The huNOG-EXL mice are modified to express human GM-CSF and IL3, allowing these mice to support human immune cells of myeloid lineage, in addition to lymphocytes. We evaluated subcutaneous tumor growth and measured metastasis in 22Rv1-engrafted huNOG-EXL mice under various levels of androgenic signaling. Mice were divided into four different conditions, listed in descending order of AR-activity outlined in Sedelaar and colleagues, 2013 (26): (i) mice castrated and implanted with testosterone to raise and maintain testosterone levels (530 ± 50 ng/dL) that are physiologically relevant in normal humans (26); (ii) mice with intact gonads, thus mimicking hypogonadal or castrate human levels; (iii) mice castrated, thus mimicking patients treated with abiraterone [the CYP17A inhibitor (27)]; and (iv) mice castrated and dosed with enzalutamide, thus mimicking androgens depleted and AR-antagonized conditions. Similar to the result in 22Rv1-engrafted huNOG mice with two different HSC donors, primary tumor growth was not affected by either castration or enzalutamide treatment (Supplementary Fig. S8). However, unlike 22Rv1-engrafted huNOG, no difference in metastasis was observed in the huNOG-EXL mice treated with enzalutamide (Fig. 4A–H), suggesting a possible role of mature myeloid cells in preventing the enzalutamide effect on metastatic growth seen in huNOG mice. This suggests the possibility of immunosuppressive myeloid cells in tumors of huNOG-EXL mice.

**Figure 5.**
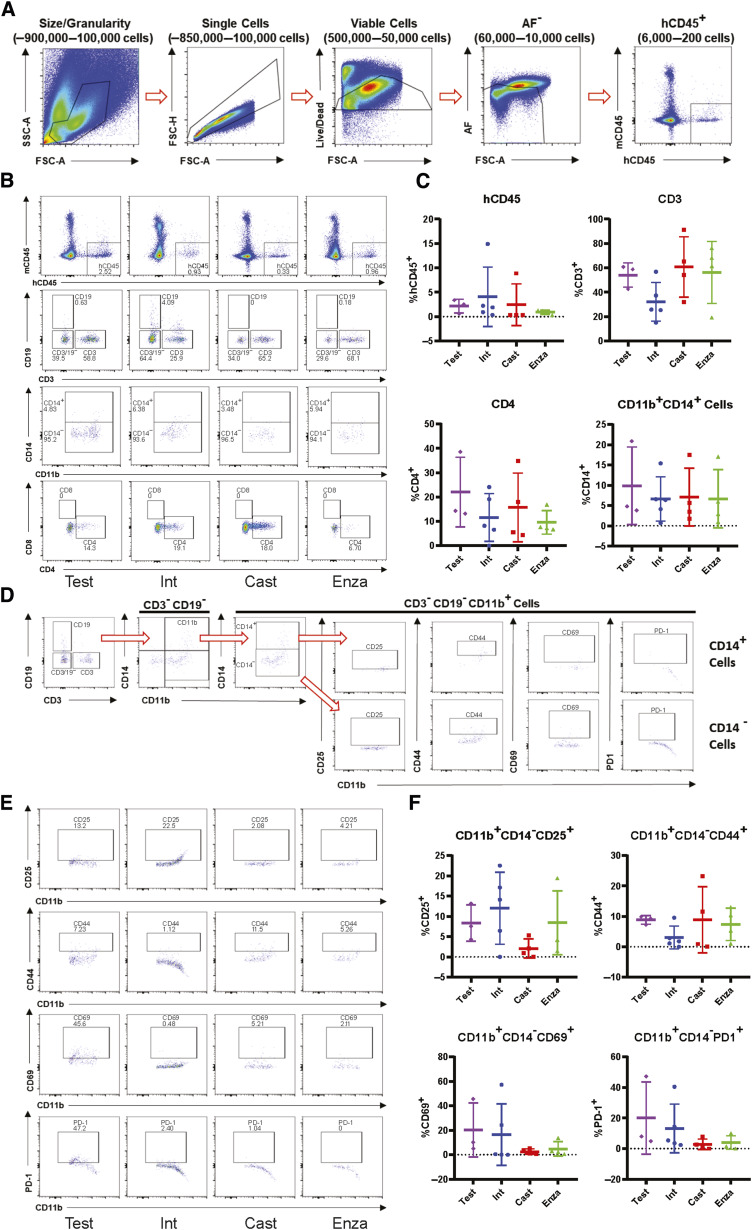
Myeloid-support exhibits immuno-dampened profile in huNOG-EXL 22Rv1 xenograft tumors. **A,** Gating strategy used to determine presence of human CD45^+^ cells in the NOG-EXL model. **B,** Representative data showing the abundance of various immune cell populations; human leukocytes, CD19^+^ cells, CD3^+^ cells, and double negative cells; MDSC and activated myeloid cells; and helper T cells (CD4^+^) and cytotoxic T cells (CD8^+^; top to bottom). **C,** Quantitated data comparing human CD45, CD3, CD4, and CD11b populations in tumors isolated from testosterone-implanted vehicle, intact vehicle, castrated vehicle, and castrated enzalutamide treated mice. **D,** Gating strategy for determining the activation state of MDSCs (CD3^−^ CD19^−^ CD11b^+^ CD14^−^). **E,** Representative data showing the activation state of the MDSC cells harvested from tumors under different treatment categories through the presence of the surface markers: CD25, CD44, CD69, and PD-1. **F,** Quantitated data showing the activation state of the MDSCs throughout the different treatments.

Our data from TILs (Fig. 3) showed that there were activated T cells in the tumors of 22Rv1-engrafted huNOG mice. We immunologically profiled subcutaneous tumors from 22Rv1-engrafted huNOG-EXL mice using the Cytek Aurora system to understand the difference in enzalutamide effect in huNOG compared with huNOG-EXL mice (See details of antibody panel in Materials and Methods). TILs from 22Rv1-engrafted huNOG-EXL mice tumors were collected for immune profiling from treatment groups with descending levels of androgenic signaling as described for Fig. 4 [either castrated/testosterone implant (Test), intact/vehicle (Int), castrated/vehicle (Cast) or castrated/enzalutamide (Enza)]. Analysis of the population of leukocytes (CD45^+^), T cells (CD3^+^), helper T cells (CD4^+^), and myeloid cells (CD3^−^ CD19^−^ CD11b^+^; Fig. 5A) showed no differences between the treatments in the intra-tumoral populations of these immune cells (Fig. 5B and C) nor any major differences within the spleens (Supplementary Figs. S9–S11) in the huNOG-EXL model, with similar levels of human leukocyte percentages in the overall tumor. The populations, and activation levels determined by CD25 [the IL2 receptor, induced upon initial T-cell activation, constitutively expressed in T-regulatory cells (Tregs; ref. 28)], CD44 [upregulated upon activation in effector and memory T-cells (29)], CD69 [a broadly expressed early activation marker in leukocytes implicated in retaining cells in peripheral tissues (30)], and PD1 [programmed cell death-1, a marker of T-cell exhaustion and Treg differentiation (31)] abundance (Fig. 5D), of CD14 negative myeloid cells also showed no differences between the treatments in this model (Fig. 5E and F). The activation level was low, suggesting the huNOG-EXL represents an immune “cold” model similar to what is seen in the majority of prostate cancer patients (32, 33). The population of CD14^+^ myeloid cells observed was too small to determine their level of activation (Fig. 5). In fact, it is interesting to note that for all treatments, the majority of myeloid cells were CD14-negative, which is indicative of immature myeloid derived suppressor cells (MDSCs; ref. 34). We identified very few tumor-infiltrating B cells (CD19 positive) and natural killer (NK) cells (CD56^+^, CD16^+^) across hormone conditions, despite high levels in the spleens of these mice (Supplementary Fig. S11E–G).

**Figure 6.**
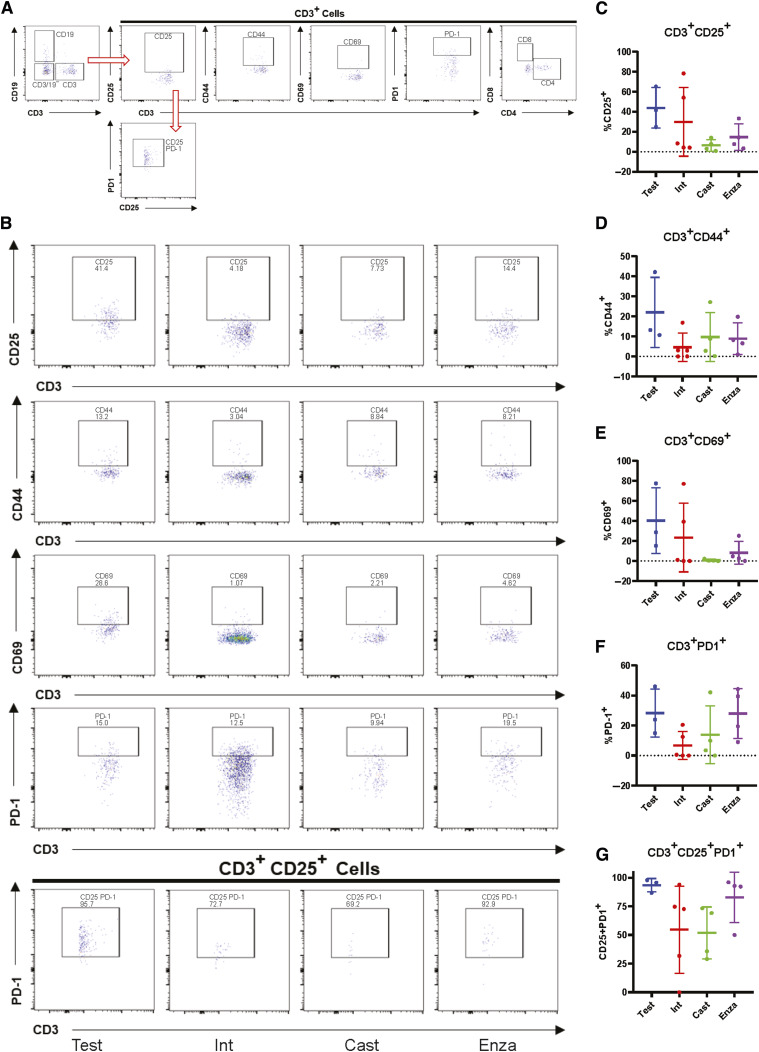
Immune-dampened profile of T cells in huNOG-EXL tumors. **A,** Gating strategy for determining the activation state of T cells and regulatory-like T cells. **B,** Representative data showing the activation state of the CD3^+^ cells harvested from tumors under different treatment categories through the presence of the surface markers: CD25, CD44, CD69, and PD-1 and regulatory-like cells (CD3^+^CD25^+^PD-1^+^). **C,** Quantitation showing the expression of CD25 (**D**), CD44 (**E**), CD69 (**F**), PD-1 (**G**) regulatory-like T cells.

We also profiled T cells from 22Rv1-engrafted huNOG-EXL mice tumors for activation markers and to determine the population of Tregs (Fig. 6A). Although no differences were observed between treatment groups (Fig. 6B–G), for the CD3^+^ CD25^+^ cells (Fig. 6C), the majority were PD1^+^ (Fig. 6F), which is indicative of the regulatory T-cell (together in Fig. 6G) phenotype found in the bone-microenvironment of prostate cancer patients (16). We confirmed these findings through multiplexed immunofluorescence (IF) and immunohistochemical (IHC) staining of tumors for CD3, CD8, and Granzyme B expression (IF-Supplementary Fig. S11) and AR and PD-1 expression (IHC-Supplementary Fig. S12). We find few CD3^+^ T cells within these tumors, even fewer CD8^+^ cells, with even fewer of them expressing the activation/effector molecule Granzyme B (35). Consistent with the flow cytometry results, we find few differences across all groups for their expression (Supplementary Fig. S12), with some slight elevation detected in one liver metastasis, and no significant differences in AR and PD-1 staining across the treatment groups (Supplementary Fig. S13), with PD-1^+^ cells detected in all tumors. This result, together with the data on MDSCs (Fig. 5) suggests a suppressed immune profile (“cold”) in the huNOG-EXL system, in contrast to the activated immune profile (“hot”) seen in the huNOG model (Fig. 3).

### VCaP xenograft tumors respond to immunotherapy and enzalutamide in the huNOG model

**Figure 7.**
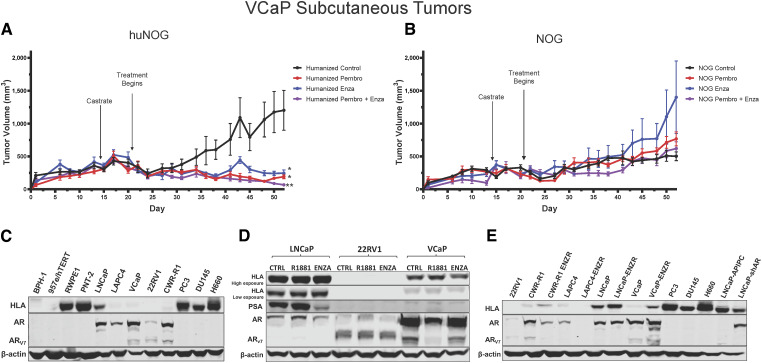
Response of VCaP subcutaneous tumors to anti-PD1 immunotherapy and enzalutamide. **A,** huNOG primary tumor growth response and (**B**) NOG primary growth response to pembrolizumab, enzalutamide, and combination therapy. *, *P* > 0.05 when compared with vehicle control, **, *P* > 0.05 when compared with all other conditions. **C,** HLA (HLA-A, B, C) expression of a panel of benign prostate (BPH-1, 957e/hTERT, RWPE1, PNT-2), AR-positive prostate cancer (LNCaP, LAPC-4, VCaP, 22Rv1, CWR-R1) and AR-negative (PC3, DU145 and NCI-H660) cancer cell lines determined by Western blotting (representative blots show, experiment replicated three times). Corresponding AR blot and AR variant (AR-V7) detection. **D,** LNCaP, 22Rv1, and VCaP HLA response the AR agonist, R1881 (1 nmol/L), AR-antagonist enzalutamide (ENZA - 10 μmol/L), and vehicle control (CTRL) treatment for 24 hours. PSA is a canonical AR-target gene as a readout of AR modulation. **E,** Comparison of HLA expression between enzalutamide naïve and resistant cell lines (ENZR).

To test another xenograft model in humanized mice, we relied on the VCaP castration-resistant-xenograft model, which mimics the natural history of prostate cancer, with short-term responses to both castration and AR antagonism (19). We assayed primary tumor growth using a VCaP castration resistant model in huNOG mice to evaluate the model in a more enzalutamide sensitive setting, determine if we could enhance the effects of immune activation by treatment with a checkpoint inhibitor in the form of the anti-PD1 pembrolizumab (pembro). In this model, VCaP tumor-bearing mice were castrated when the tumors were approximately 200 mm^3^ in size, and once the tumor grew back, animals were randomized and treated with enzalutamide and/or the anti-PD-1 antibody pembro, or with vehicle controls. VCaP xenograft tumors treated with enza and pembro work well as monotherapies, and slightly better in combination, completely eliminating tumors (Fig. 7A). NOG mice were not responsive to either of these treatments (Fig. 7B). This result demonstrates that this model is responsive to immune-checkpoint inhibition and works synergistically with AR-targeted therapy.

Finally, to provide some insight in the mechanism of the anti-tumor effects seen in the huNOG mice and to identify Androgen-control of HLA (A, B, C) expression, we assayed prostate cancer cells for HLA expression (Fig. 7C). 22Rv1 cells display no expression of HLA molecules, regardless of AR modulation (Fig. 7C–E); however, VCaP cells illustrated increased HLA expression in the enzalutamide-resistance state (Fig. 7D), and slight decreases with short-term treatment (Fig. 7E). Other AR-positive prostate cancer cell lines generally display decreases in HLA expression when compared with immortalized benign controls (Fig. 7C), and cells that maintain AR expression after enza-resistance show increased HLA expression, as evidenced by previous reports (2, 33, 36, 37). Taken together, these data suggest that much of this response is likely governed by the T-cell activation seen with enza treatment (38), as well as some potential cancer cell autonomous effects, all of which are topics for future investigation.

## Discussion

To the best of our knowledge, our results illustrate that the first model of human prostate cancer that metastasizes to clinically relevant locations has an intact human immune system, and responds appropriately to the standard-of-care hormonal therapies. We found that humanizing tumor-immune interactions improved modeling of metastatic prostate cancer and provides a model more suitable to evaluate hormonal- and immunotherapies. These huNOG mice provide a model that not only has an intact human immune system, but also shows metastases to relevant secondary sites and models the effect anti-androgen treatment has on metastasis. In 22Rv1 xenografts, subcutaneous tumor size was not significantly altered across hormone conditions; however, metastasis to secondary sites differed in castrate huNOG mice treated with enza versus background-matched immunocompromised mice. VCaP xenograft tumors showed decreases in growth with enza and anti-Programed-Death-1 treatments in huNOG mice, with combination eliminating tumors. No effects were seen with any treatment in NOG mice. Enza responses in huNOG and NOG mice were distinct and associated with increased T cells within tumors of enza-treated huNOG mice, and increased T-cell activation. In huNOG-EXL mice, which support human myeloid development, there was a strong population of immunosuppressive regulatory T cells and MDSCs, and enza treatment showed no difference in metastasis.

The 22Rv1-engrafted huNOG mouse models human prostate cancer in that it metastasizes to clinically relevant locations. Metastasis was observed in bone (femur and humerus), lymph nodes, liver, brain, spleen, lungs, and kidneys (adrenal) in 22Rv1-engrafted huNOG, as well as in the immunocompromised NOG mice. However, reduction of metastasis by enzalutamide was only observed in the huNOG mice. In the immunocompromised NOG mice, enzalutamide treatment did not decrease metastasis, but instead, it showed a paradoxical increase in metastases. The decreased metastasis with enzalutamide treatment in the huNOG mice is consistent with the clinical situation where Hussain and colleagues (20) found that enzalutamide treatment of patients with non-metastatic CRPC had a 71% lower risk of metastasis. In this way, the xenograft model in huNOG mice has the advantage in that it responds appropriately to standard-of-care hormonal therapies.

An important feature of this xenograft model in huNOG mice is the presence of tumor-immune interactions. The huNOG mice maintain cells of the human immune system, including functioning human T cells. Taken together, the results suggest that the effect of enzalutamide to decrease metastasis involves an interaction with the immune system. This hypothesis is supported by the presence of increased infiltration of activated (INFγ^+^) T cells into the tumors from the huNOG mice treated with enzalutamide, but surprisingly, no differences in TNFα were observed (Supplementary Fig. S6). As androgen signaling has been shown to be immunosuppressive (38, 39), AR antagonism might relieve an inhibitory signal and activate immune surveillance, as well as promote T-cell tumor infiltration. These results suggest that a huNOG xenograft model exhibits an activated “hot” immune profile from an immune perspective, and this may be important in the ability of the model to replicate the action of enzalutamide to decrease metastasis as seen in the clinical situation.

Interestingly, enzalutamide’s ability to decrease metastasis was not observed in the huNOG-EXL model. The huNOG-EXL mice maintain human immune cells of both a myeloid and lymphoid lineage. The TILs from tumors from enzalutamide treated huNOG-EXL mice did not show the increased infiltration of activated T cells that was observed with the huNOG mice, and the presence of MDSC-like cells and Tregs suggests a suppressed “cold” immune profile in the huNOG-EXL system. This immunosuppressed profile may account for the lack of effect of enzalutamide on metastasis in this system. Enzalutamide itself has been shown to promote immunosuppressive effects in myeloid populations through a non-AR dependent manner (40).

The huNOG mouse xenograft model can be used for other prostate cancer tumor cell lines, besides 22Rv1. Using a VCaP castration resistant model in huNOG mice, we demonstrated the usefulness of a huNOG mouse xenograft to evaluate immunotherapies, either as monotherapies or in combination with a hormone treatment such as enzalutamide. VCaP xenograft tumors were responsive to the anti-PD1 pembrolizumab as a monotherapy, and in combination with enzalutamide, they worked synergically (Fig. 7A). Tumors in immunocompromised NOG mice were not responsive to either of these treatments (Fig. 7B).

Future work will examine how AR and its signaling exhibits the cell-specific effects within prostate tumors and how this affects interactions between cancer cells and immune cells in the tumor microenvironment to produce differential responses to AR-targeted therapies. One important question is the role of PD-1 expression, or the presence of Tregs in the huNOG model. Unfortunately, the low overall abundance of T cells compared with 22Rv1 tumor cells in the huNOG subcutaneous xenografts limited our ability to reliably assay for Tregs by flow cytometry, and histological assessments were similarly unreliable. However, this interesting concept should be explored further due to the dominant role that T cells play in this model. Additionally, we observed that enzalutamide resistant cells upregulate HLA, here (Fig. 7E) and as previously reported (2), but others have also illustrated a connection between chronic enzalutamide treatment and upregulation of immune-regulatory proteins that produce an immunosuppressed microenvironment (41). Our models have the potential to aid further work that is necessary to tease apart the timing and phasing of AR inhibition to produce an optimal immune response.

The huNOG mice illustrate decrease metastatic burden with enzalutamide, even with a resistant cell line, and may even show stronger effects in the bone versus visceral organs, which is also observed clinically (42). However, while this better represents what is observed clinically, the huNOG-EXL model better mirrors a typical “cold” prostate immune microenvironment. This is an interesting discrepancy and may have to do with the sites of the primary tumors verses metastases or even how the AR-targeted therapies change HLA expression. One potential future approach to expand on the results of this study is to assess this model using an orthotopic prostate injection rather than a subcutaneous injection of the prostate tumor cells. This would be even more relevant in huNOG-EXL xenografts, given that xenograft tumor site shows differential effects to myeloid and immune-checkpoint responses in other tumor types (43), and could provide an approach with a more relevant immune microenvironment. Future studies will also focus on the role of AR in these models, in both the stroma, the cell-autonomous signaling, and tumor-stroma interactions, even in AR-negative prostate cancer cells, which seem to express higher levels of HLA when compared with AR-positive disease (Fig. 7).

Excitingly, for a subset of patients, use of biphasic androgen therapy has produced responses to immune-checkpoint inhibition (44), which we can also model in our huNOG systems. Finally, we illustrated the use of this model across several cancer cell line derived xenografts; however, we hope to utilize mice with humanized immune systems to model the diverse clinical responses seen with AR- and immune-checkpoint inhibition in other tumor models. Particularly those from patient derived xenografts and organoids, such as CDK12 mutated tumors–some of which are responsive to checkpoint inhibition (45)–and to model clinical resistance to different therapies and disease states that otherwise has been difficult to do with standard xenografts or GEM models.

## Supplementary Material

Figure S1 Representative histology of subcutaneous tumor, bone and liver metastasis of 22Rv1 cells in huNOG mice.

Figure S2 Metastasis by 22Rv1 to the humerus in huNOG and huNOG-EXL mice.

Figure S3 Metastasis by 22Rv1 to the skull in huNOG and huNOG-EXL mice.

Figure S4 Metastasis by 22Rv1 to the spleen in huNOG and huNOG-EXL mice.

Figure S5 Metastasis by 22Rv1 to the lung in huNOG and huNOG-EXL mice.

Figure S6 Metastasis by 22Rv1 to the heart in huNOG and huNOG-EXL mice.

Figure S7 Percentage of CD3+ cells showing TNF-α expression via intracellular staining isolated from subcutaneous 22Rv1 tumors huNOG mice.

Figure S8 22Rv1 growth in huNOG-EXL and NOG mice.

Figure S9 Immune-profile in huNOG-EXL 22Rv1 xenograft spleen.

Figure S10 Immune-profile of T-cells in huNOG-EXL spleen.

Figure S11 Immune-profile of spleen and tumor B-cell Populations and B-cell activation NK-Cell population in huNOG-EXL spleen.

Figure S12 Multi-IF for CD3, CD8 and Granzyme B of 22Rv1 tumors in huNOG-EXL mice.

Figure S13 IHC of AR and PD1 of 22Rv1 tumors in huNOG-EXL mice.

Supplementary Figure Legends Supplementary Figure Legends
