# Clinically relevant humanized mouse models of metastatic prostate cancer facilitate therapeutic evaluation

**DOI:** 10.1158/1541-7786.MCR-23-0904

**Published:** 2024-09-04

**Authors:** Raymond J. Kostlan, John T. Phoenix, Audris Budreika, Marina G. Ferrari, Neetika Khurana, Jae Eun Choi, Kristin Juckette, Somnath Mahapatra, Brooke L. McCollum, Russell Moskal, Rahul Mannan, Yuanyuan Qiao, Donald J. Vander Griend, Arul M. Chinnaiyan, Steven Kregel

**Affiliations:** 1 Department of Cancer Biology, Loyola University Chicago, Maywood, IL 60153; 2 Integrated Program in Biomedical Science, Biochemistry, Molecular and Cancer Biology, Loyola University Chicago, Maywood, IL, USA.; 3 Michigan Center for Translational Pathology, University of Michigan, Ann Arbor, MI, USA; 4 Department of Pathology, University of Michigan, Ann Arbor, MI, USA; 5 Rogel Cancer Center, University of Michigan, Ann Arbor, MI, USA; 6 Department of Pathology, University of Illinois at Chicago, Chicago, IL, USA

## Abstract

There is tremendous need for improved prostate cancer (PCa) models. The mouse prostate is anatomically and developmentally different from the human prostate and does not spontaneously form tumors. Genetically engineered mouse models lack the heterogeneity of human cancer and rarely establish metastatic growth. Human xenografts are an alternative but must rely on an immunocompromised host. Therefore, we generated PCa murine xenograft models with an intact human immune system (huNOG and huNOG-EXL mice) to test whether humanizing tumor-immune interactions would improve modeling of metastatic PCa and the impact of androgen receptor-targeted and immunotherapies. These mice maintain multiple human immune cell lineages, including functional human T-cells and myeloid cells.

## Introduction

Prostate cancer is one of the leading causes of cancer-related deaths, and treatment options for men with advanced, metastatic disease are limited. Progression to metastatic castration resistant prostate cancer (mCRPC) is often driven by the maintenance of androgen receptor (AR) signaling despite attempted blockades with standard-of-care treatments such as castration and enzalutamide (enza) (1,2). Development of novel therapeutics for mCRPC depends on improving the murine models of this disease. The current genetically engineered model (GEM) systems for mCRPC exhibit several shortcomings that hinder their clinical applicability. GEM systems rely on the murine prostate, which differs anatomically and developmentally from the human prostate, and does not form sporadic tumors (3,4). GEMs lack the heterogeneity of human disease and rarely establish metastatic growth, and disease progression in GEM systems tend to be driven in a contrived manner unrelated to human disease or the commonly observed drivers of disease progression (5–7). While human xenografts represent alternative models, they rely on tumor growth in an immunocompromised murine host and are thus unsuitable for investigations into tumor-immune interactions and immunotherapy interventions, a rapidly expanding area of cancer research (5).

Consequently, the development of a human-derived model that can recapitulate the natural history of the disease—from initiation to metastatic spread—and will respond appropriately to the standard of care hormonal therapies is needed to accelerate translational progress in prostate cancer research (5–7). To address this issue, we employed a series of prostate cancer xenograft models in recently developed murine lineages with an intact human immune system (huNOG and huNOG-EXL mice, Taconic Biosciences, Germantown, NY). Male huNOG mice are produced by engrafting juvenile immunocompromised NOG (NOG-NOD/SCID/γnull/c) mice with human CD34+ hematopoietic stem cells (HSC) from human umbilical cord blood. These mice stably develop and maintain multiple human cell lineages, including functional human T-cells, and can be human leukocyte antigen (HLA) matched for a variety of xenograft models (8). Additional mouse models produced with human cytokine Interleukin-3 (IL-3) and Granulocyte-macrophage colony-stimulating factor (hGM-CSF)] huNOG-EXL, provide more support to human myeloid cells which are often outcompeted by the host in standard huNOG mice (8,9). In the context of enza treatment, we revealed differential levels of metastatic outgrowth in NOG immunocompromised controls compared to newly developed huNOG mice. These results led us to hypothesize that the anti-metastatic responses of AR-targeted therapies are achieved through the human immune system and prompted us to profile the differences in immune microenvironment populations across our models. Here we describe new tools to assay the therapeutic responses of metastatic PCa xenografts in huNOG and huNOG-EXL, which model an activatable T-cell and an immunosuppressed subcutaneous tumor microenvironment, respectively.

## Methods

### Cell lines and culture:

R1881 was purchased from Sigma-Aldrich (St. Louis, MO), and enzalutamide (MDV3100), and anti-Programmed Death-1 [anti-PD1, Pembrolizumab (Pembro)] were purchased from Selleck Chemicals (Houston, TX), and stored at −20°C in ethanol, −80°C in DMSO, and constituted fresh from lyophilized powder in PBS, respectively. CWR-22Rv1 (22Rv1), and VCaP cell lines were purchased from American Type Culture Collection (Manassas, VA) and were validated and cultured as described (2,10), and transduced with lentivirus for luciferase (luc2) as previously described (2). CWR-R1, VCaP, LAPC-4, LNCaP, and enzalutamide-resistant counterparts, in addition to BPH-1, 957E/hTERT, NCI-H660 (H660), PC3, DU145, PNT-2, and RWPE1 cells, were generously provided by Dr. Donald J. Vander Griend at the University of Illinois at Chicago and have been previously characterized and cultured as described (2,11,12). Dr. Peter Nelson at Fred Hutchinson Cancer Center provided LNCaP-sh and LNCaP-APIPC cells (13). All cultures were routinely screened for mycoplasma contamination using the ATCC Universal Mycoplasma Detection Kit (Manassas, VA).

### Murine prostate tumor xenograft models:

NOG control, huNOG and huNOG-EXL humanized mice were obtained from Taconic Biosciences (Germantown, NY). huNOG and huNOG-EXL came engrafted from two different independent HSC donors, and an N=3 for each donor and control per condition to maintain minimal statistical power and illustrate reproducibility. Mice were anesthetized using 2% Isoflurane (inhalation) and either 1×10^6^ VCaP or 5×10^5^ 22Rv1 cells suspended in 100 μL of PBS with 50% Matrigel (BD Biosciences) were implanted subcutaneously into the dorsal flank on both sides of the mice. Once the tumors reached a palpable stage (100 mm^3^), the animals were randomized and treated with enzalutamide or vehicle control [1% Carboxymethylcellulose (Sigma Aldrich), 0.25% TWEEN-80 (Sigma Aldrich), and 98.75% PBS] by oral gavage. Growth in tumor volume was recorded using digital calipers and tumor volumes were estimated using the formula (π/6) (*L* × *W*^2^), where *L* = length of tumor and *W* = width. Body weight during the course of the study was also monitored. At the end of the studies, once the largest tumors met defined endpoints of 1 cm^3^ (22 days, post injection for 22Rv1 cells), mice were sacrificed, tissues imaged *ex-vivo*, and tumors were extracted for the downstream analyses. For testosterone implantation, mice were surgically castrated and concurrently implanted with silastic tubing containing 25 mg testosterone (Steraloids Inc, Newport, RI) for sustained release. 22Rv1 cell implantation occurred after 1–1.5 weeks of allowing circulating testosterone levels to equilibrate to approximate human hormone levels (14).

For the VCaP-CRPC experiment, VCaP tumor bearing mice were castrated when the tumors were approximately 200 mm^3^ in size after 14 days. Once the tumor grew back to the pre-castration size, 7 days later, the animals were treated with either vehicle (oral gavage or PBS intraperitoneal injection), enzalutamide (10 mg/kg 5 days a week via oral gavage), pembrolizumab (1 mg/kg 3 times a week, intraperitoneal injection), or both, with respective controls. Mice were sacrificed at endpoint when tumors approached 1 cm^3^, which was 52 days after initial injection. All procedures involving mice were approved by the University Committee on Use and Care of Animals (UCUCA) at the University of Michigan or Loyola University Chicago and conform to all regulatory standards.

### *Ex-vivo* imaging:

At tumor endpoint, mice were injected with 150 mg/kg body mass D-luciferin (Promega) via intraperitoneal injection, then they were humanely sacrificed, mice necropsied, and tissues were rapidly imaged (within 10 minutes post-sacrifice) with the bioluminescence signal being assessed with the IVIS Spectrum In Vivo Imaging System (PerkinElmer). Tissue tumor burden was calculated based on the total flux (photons per second [p/s]) normalized to area (average radiance), utilizing Living Image software and statistics performed using Graph Pad Prism by utilizing ANOVA with multiple testing corrections across samples and Mann-Whitney and Kolmogorov-Smirnov t-tests between groups.

### Flow Cytometry:

#### huNOG experiments:

After *ex vivo* imaging, mononuclear cells were isolated from the subcutaneous tumors and spleen and were stained with fluorescently conjugated antibodies as previously described (15). CountBright Absolute Counting Beads (Thermo Fisher) were used to quantify cell number. For cytokine staining, lymphocytes were incubated at 37 °C for 4 hours in culture medium containing PMA (5 ng mL−1), ionomycin (500 ng mL−1), Brefeldin A (1:1,000) and Monensin (1:1,000). Extracellular staining was performed for 20 minutes using the antibodies listed below, cells were then washed and resuspended in 1 mL of freshly prepared Fix/Perm solution (BD Biosciences) and incubated overnight at 4 °C. The cells were then washed Perm/Wash buffer (BD Biosciences) and stained with intracellular antibodies listed below. Data collection and analysis was performed on a LSRII equipped with four lasers or a Fortessa equipped with four lasers (BD Bioscience) using BD FACS Diva software. The following human antibodies were used: CD45 (BD Biosciences), CD3 (Thermo Fisher Scientific), CD8 (Biosciences), CD4 (Thermo Fisher Scientific), and IFN-γ (BD Biosciences). All antibodies were used at a 1:100 dilution, as previously described (16).

#### huNOG-EXL experiments:

Similar to above, however we utilized the Cytek 5 Laser Aurora ^®^ full spectrum flow cytometer (Cytek Biosciences, Bethesda, MD). Tumor and spleen samples were frozen in liquid nitrogen and later thawed and harvested to acquire mononuclear cells. After harvesting, cells were aliquoted and resuspended in Human TruStainFcX (Clone Information Proprietary, BioLegend) and TruStain FcXPlus (Clone S17011E, BioLegend) according to the provided recommendation (5 μLper 100 μL PBS per 1 million cells). Cells were then washed and stained in L/D Fixable Dead Cell Stain (Thermo Fisher Scientific) for 30 min. Next cells were washed, and extracellular staining was performed using a panel of antibodies listed below for 30 min. Data collection was performed using SpectroFlo 3.0.1. The following antibodies were used: mCD45 (Clone 30-F11, BD Biosciences) hCD45 (Clone HI30, BioLegend), CD3 (Clone UCHT1, BioLegend), CD4 (Clone RPA-T4, BioLegend), CD8a (Clone RPA-T8, BioLegend), CD11b (Clone TCRF44, BioLegend), CD14 (Clone 63D3, BioLegend), CD16 (Clone B13.1, BioLegend), CD19 (Clone HIB19, BioLegend), CD25 (Clone BC96, BioLegend), CD44 (Clone IM7, BioLegend), CD56 (Clone 5.1H11, BioLegend), CD69 (Clone FN50, BioLegend) PD-1 (Clone NA105, BioLegend). Antibodies were titrated for optimal fluorescence per 1 million cells.

### Multiplex immunofluorescence (multi-IF):

Multiplex immunofluorescence (multi-IF) was conducted on 4-μm-thick tissue sections that were whole formalin-fixed and paraffin-embedded (FFPE). Anti-CD3 rabbit monoclonal antibody (Ventana, catalog no. 790–4341), anti-CD8 rabbit monoclonal antibody (Ventana, catalog no. 790–4460), and anti-granzyme rabbit polyclonal antibody (Cell Marque, catalog no. 262A-1b) were used. The multiplexing process was carried out consecutively in a sequence of granzyme, CD8, and CD3 with heat denaturation before the second primary antibody incubation. The process was automated using the Discovery Ultra automated slide staining system (Roche-Ventana Medical Systems), and OmniMap anti-rabbit and anti-mouse HRP kits (Ventana, catalog no. 760–4311 and 760–4310) were used. Discovery Cy5 Kit (RTU, Roche-Ventana Medical Systems, catalog no. 760–238) was used for granzyme, Discovery Red 610 (RTU, Roche-Ventana Medical Systems, catalog no. 760–245) for CD8, and Discovery FITC (RTU, Roche-Ventana Medical Systems, catalog no. 760–232) for CD3 were the fluorophores used. The staining was independently assessed by two study pathologists (RM and SM) at ×100 and ×200 magnification to check for the presence and pattern of expression.

### Immunohistochemistry and histology:

Immunohistochemistry was performed on whole formalin-fixed, paraffin-embedded (FFPE) 4-μm-thick tissue sections using anti-AR rabbit monoclonal antibody (Ventana, catalog no. 790–4605) and anti-PD1 mouse monoclonal antibody (Ventana, catalog no. 760–7894). Heat-induced epitope retrieval was used, along with conditioning media (CC1) and signal development by OmniMap anti-rabbit (Ventana, catalog no. 760–4311) and anti-mouse HRP kits (Ventana, catalog no. 760–4310). The presence or absence of a brown signal in the nucleus and cytoplasm within the tumor was assessed by the study pathologists (RM and SM). Hematoxylin and Eosin staining of tumors and metastases performed as previously described (2).

### Western blotting:

Whole-cell lysates collected from cells seeded at 1×10^6^ cells per well of a 6 well plate (Becton, Dickinson and Company, Franklin Lakes, New Jersey), were lysed in RIPA-PIC buffer [150 mM sodium chloride, 1.0% Igepal CA-630 (Sigma-Aldrich), 0.5% sodium deoxycholate, 0.1% SDS, 50 mM Tris, pH 8.0, 1× protease inhibitor cocktail (Roche Molecular Biochemicals; Penzberg, Germany)], scraped, and sonicated (Fisher Scientific; Hampton, NH; model FB-120 Sonic Dismembrator). Protein was quantified by BCA assay (Thermo-Fisher Scientific), and 50μg of protein were loaded per lane. Antibodies used were: anti-AR (D6F11 XP ^®^, Cell Signaling Technology, Danvers, MA); anti-Beta Actin (AC-15, Sigma-Aldrich); pan-anti-HLA -A-B-C (HLA class I ABC Polyclonal antibody,15240–1-AP, Proteintech group, Rosemont, IL); and anti-PSA (*KLK3*) (D11E1 XP ^®^, Cell Signaling Technology). Secondary antibodies and nitrocellulose membranes from LICOR (Lincoln, NE) were used and data captured using a LICOR Odyssey M system (Lincoln, NE) as previously described (12).

### Data availability:

All primary data, including raw and unprocessed data and images, are available upon request.

## Results

### Primary tumor and metastasis assays reveal 22Rv1-engrafted huNOG mice better reflect patient response to castration and enzalutamide treatment than NOG control mice.

**Figure 1:**
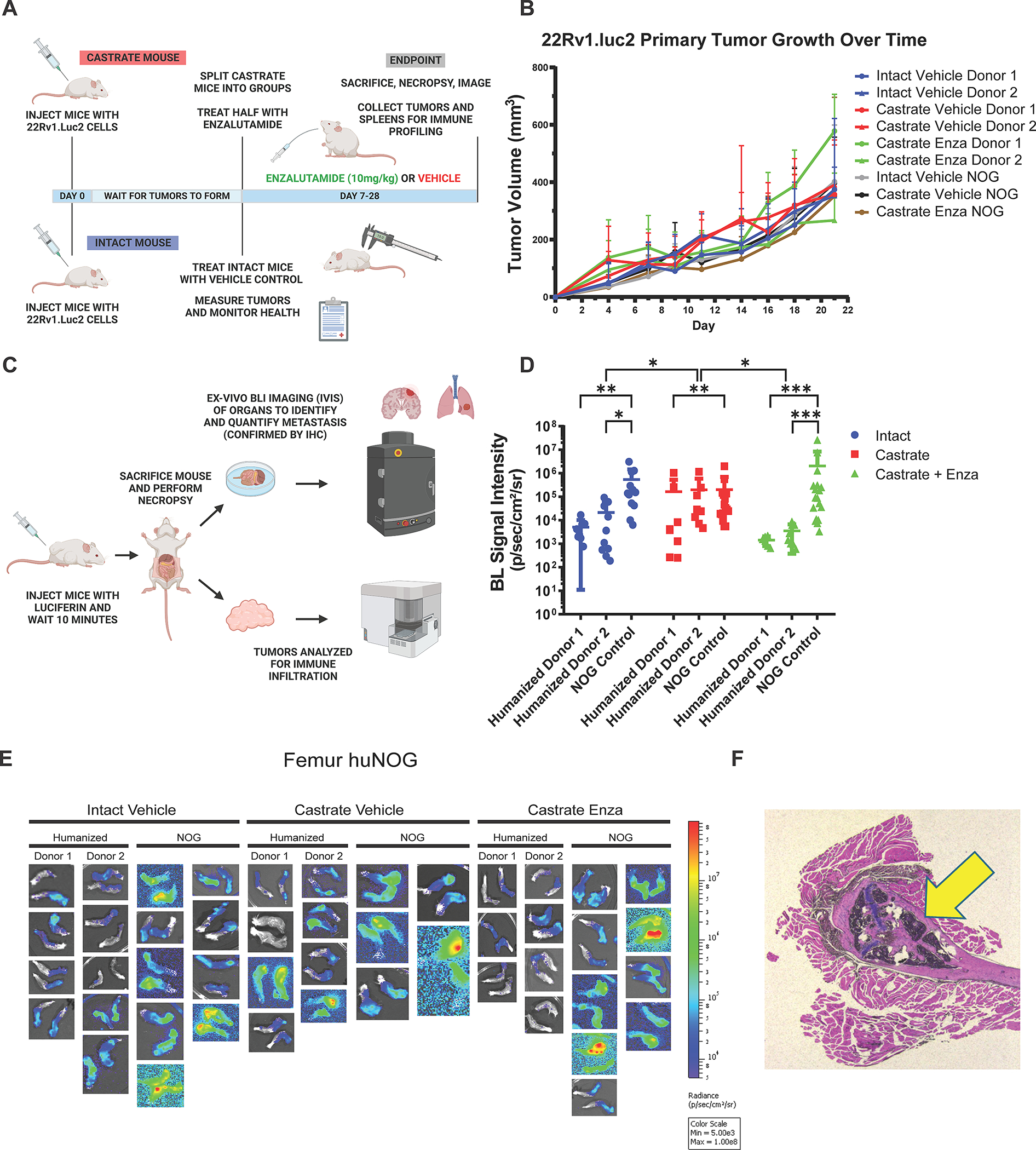
Experimental set-up and 22Rv1 growth in huNOG mice. **A,** Experimental schematic: HuNOG and NOG control mice were surgically castrated. One week following castration, control intact and castrated mice were injected subcutaneously with luciferase-transduced 22Rv1 human prostate cancer cells to assay organ-specific metastatic growth Castrated mice were then randomized and treated with enzalutamide or vehicle control (intact mice also treated with vehicle). Primary tumors were measured every 2–3 days until endpoint. huNOG: Intact n=9, Castrated n=8, Castrated Enza n=7. NOG: Intact n=9, Castrated n=5, Castrated Enza n=9. Created with BioRender.com. **B,** Subcutaneous primary flank tumor volume growth measured over time. huNOG: Intact n=18, Castrated n=16, Castrated Enza n=14. NOG: Intact n=18, Castrated n=10, Castrated Enza n=18. **C,** Schematic of end-point analysis: Prior to sacrifice, mice were injected with luciferin. At sacrifice, organs were ex-vivo analyzed for metastatic growth using the IVIS bioluminescence system (PerkinElmer), images illustrate signal intensity and location. Created with BioRender.com. **D,** Quantification of average signal intensity per unit area of bioluminescence of mouse femurs. **E,** Representative IVIS images of 22Rv1 metastasis to femur. **F,** Histological validation (H&E stain) of the femoral metastases confirmed by a pathologist (Dr. Rahul Manan, MD), with cancer cells seen in both the bone marrow and matrix of the epiphyseal head of a mouse femur (yellow arrow indicates 22Rv1 tumor mass).

**Figure 2:**
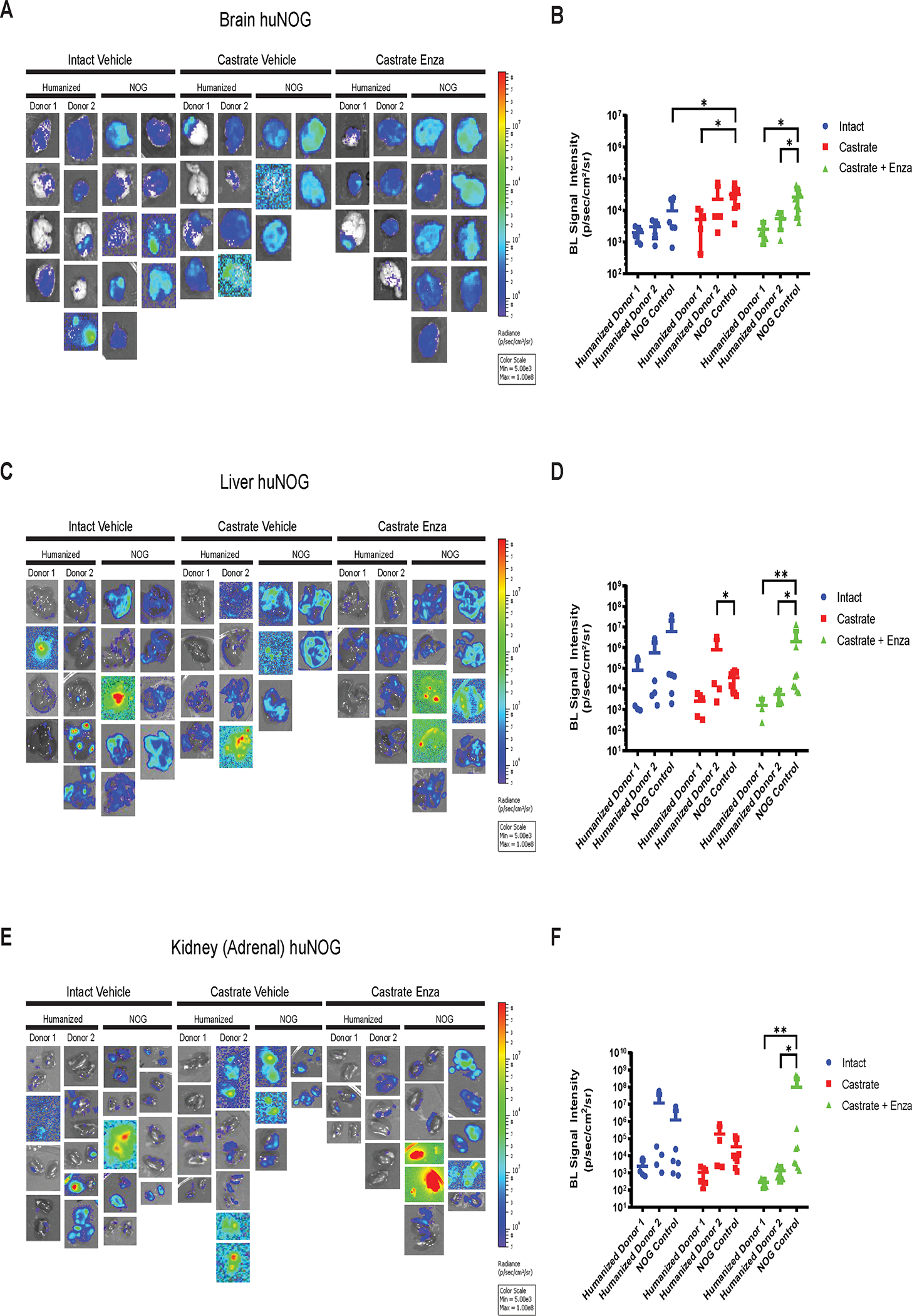
Metastasis by 22Rv1 to additional clinically relevant organs in huNOG mice. **A,** Bioluminescent images taken of mouse brain, **B,** Quantified bioluminescence of brain metastasis. **C,** Bioluminescent images taken of mouse liver, **D,** Quantified bioluminescence of liver metastasis. **E,** Kidney (adrenal gland) bioluminescent images, **F,** Quantified bioluminescence of Kidney (tumor mostly in adrenal gland).

We first sought to test the growth of the aggressive, bone metastatic CWR-22Rv1 (22Rv1) cell line (2) in huNOG mice compared to NOG [NOG-NOD/SCID/γnull/c (17)] controls with increasing levels of androgen-deprivation via surgical castration and surgical castration with enza treatment (10mg/kg- MDV3100, Selleck Chemical). 22Rv1 are the most aggressive prostate cancer cell line *in vivo* that still maintains AR expression. They express wild-type full length AR, as well as H875Y mutant, and many splice variants including V7, some of which are stably through genetic alterations (18). 22Rv1 are considered enzalutamide resistant and respond weakly to both anti-androgens and androgens (19). This cell line was produced from a xenograft made from the primary tumor of a patient with extensive bone metastases upon disease presentation. In mice, these cells metastasize and colonize clinically relevant sites such as the bone, liver, lungs, and brain (2). We assayed “primary” tumor growth via subcutaneous flank injection (Figure 1A–B) and performed metastasis detection and quantification (Figure 1C–E, Figure 2), with histological validation [representative image in Figure 1F, higher resolution images of hematoxylin and eosin (H&E) stains of subcutaneous tumors, femur and liver metastasis in Figure S1], from luciferase-tagged 22Rv1.luc2 [22Rv1 cells transduced with Promega (Madison, WI) luciferase2 ^®^ for bioluminescent imaging] to assay organ-specific metastatic growth (images of the femurs shown in Figure 1E, see Figure 2 for brain, liver and kidneys, Figure S2 for humerus, Figure S3 for skull, Figure S4 for spleen, Figure S5 for lung, Figure S6 for heart), of huNOG (two different human male CD34+ HSC donors) and NOG control mice.

The subcutaneous “primary” tumor growth was affected neither by the castration, nor enzalutamide treatment in NOG or huNOG mice at endpoint, but there was variability in presence of an intact immune system in tumor size (Figure 1B). At sacrifice, organs were ex-vivo analyzed for metastatic growth using the IVIS bioluminescence system (schematic in Figure 1C), and strikingly, metastatic growth was inhibited in huNOG mice under the conditions of castration in combination with enzalutamide treatment, (Figure 1–2) mirroring clinical responses which suggest metastatic outgrowth of castration-resistant prostate cancer can be attenuated by enza treatment (20). Surprisingly, AR antagonism with enza in immunocompromised NOG mice, contrary to clinical observations, yet observed in other models (21,22), illustrated increased metastatic colonization and growth (Figure 1D). Histology (H&E stain) confirmed femoral metastases (Figure 1F), with cancer cells seen in both the bone marrow and matrix of the epiphyseal head of a mouse femur (Figure 1F). The effect on growth was also observed for brain (Figure 2A–B), liver (Figure 2C–D) and kidney/adrenal (Figure 2E–F) metastasis in the castration/enzalutamide group for the huNOG mice. These data suggest that 22Rv1 cells have the capacity to metastasize, with or without the presence of an intact human immune system, but the immune system may be activated to prevent outgrowth at secondary sites. These data also suggest that huNOG mice better recapitulate the patient castration/enzalutamide response and warrant further investigation into the immune populations that may be responsible for this phenotype.

### Profiling T-cells in the tumors of huNOG mice reveals an activated immune profile.

**Figure 3:**
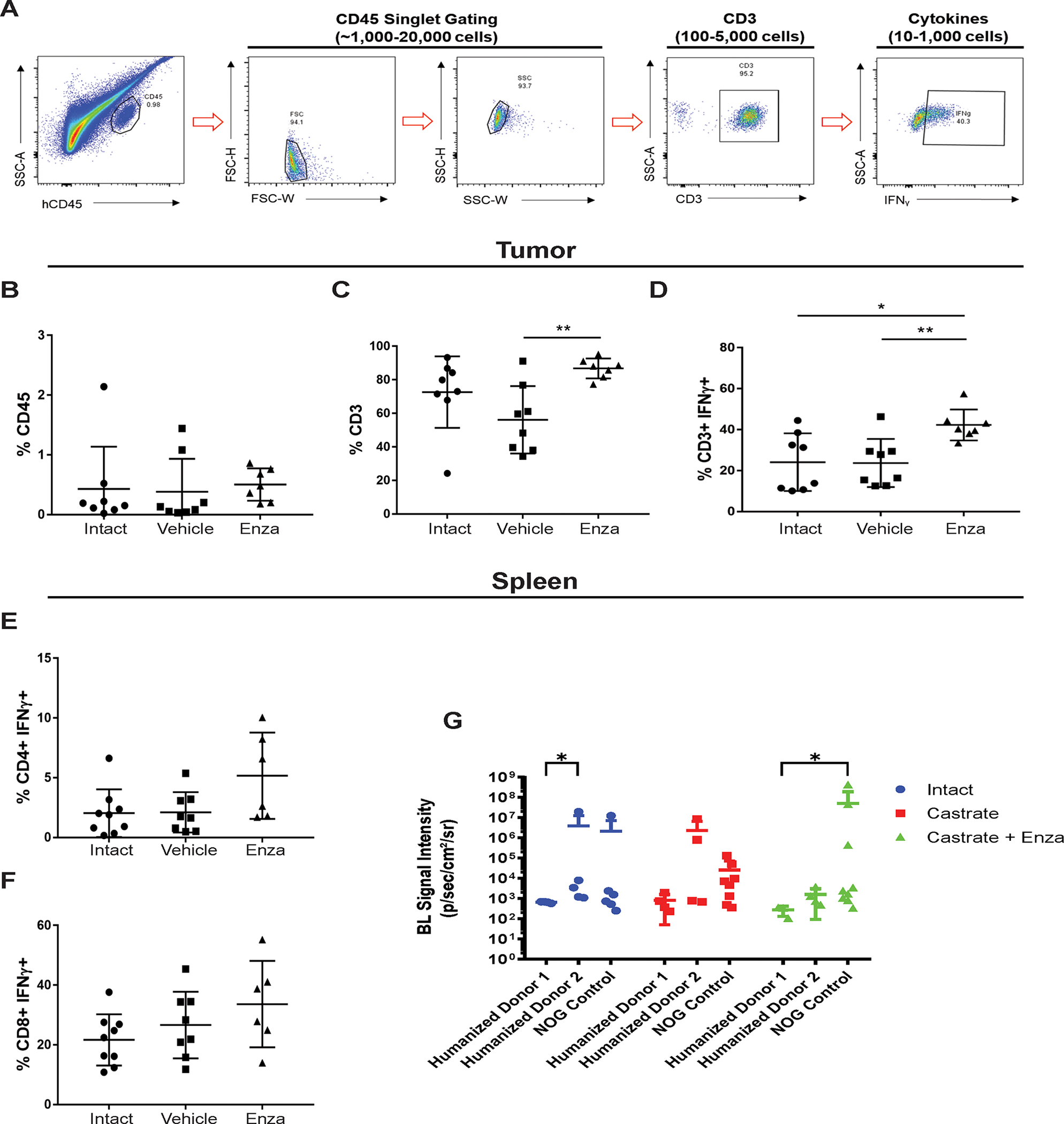
Activated T-cell immune profile in huNOG tumors. **A,** Gating strategy employed to determine T-cell activation status. **B,** Total percentage of CD45+ cells acquired from tumor tissue harvesting split between the intact control, castrated vehicle and castrated with enzalutamide treatment. **C,** Percentage of CD3+ cells observed in the total CD45+ population. **D,** Percentage of CD3+ cells showing IFNγ expression via intracellular staining. **E,** Measurement of the percentage of CD4+ cells expressing IFNγ harvested from the spleen. **F,** Measurement of CD8+ cells expressing IFNγ from the spleen. **G,** Spleen bioluminescent metastasis signal quantification.

Since 22Rv1-engrafted huNOG mice exhibited a significant decrease in metastatic colonization compared to control NOG mice (Figure 1B), we hypothesized that tumor-infiltrating lymphocytes (TILs) in huNOG mice may be responsible for this suppression, given their anti-tumor effects in patients (23,24). To evaluate TILs in 22Rv1-engrafted huNOG mice, tumors and spleen were collected for immune profiling from treatment groups as described in Figure 1A (either castrate or intact, with the castrate group further divided into enzalutamide treated or untreated). Disassociated tumors from the 22Rv1-engrafted huNOG mice were stained with human α-CD45 (for Leukocytes) and α-CD3+ (T-cells) were co-stained for intracellular IFN-γ (marker of activation), and positively gated for FACS analysis (Figure 3A). Analysis of the percentage of CD45+ leukocytes (Figure 3B), CD3+ T-cells (Figure 3C), and activated T-cells (CD3+ IFN-γ+) (Figure 3D) or (CD3+TNF-α+) (Figure S7)showed that there were significant increases in the intra-tumoral CD3+ T-cells and in their activation state (CD3+ IFN-γ+, but not TNF-α+) in the mice treated with enzalutamide in the castrated group. Overall, the percentage of human CD45+ immune cells in the tumor was low (Figure 3B), and T-cells represented the vast majority of the immune cells in the tumor (Figure 3C) in the huNOG model; thus, we focused our analysis solely on the CD3+ population. There were no significant differences in the number of splenic CD4+ helper T-cells (Figure 3E) and CD8+ cytotoxic T-cells (Figure 3F) for each of the different hormonal and enzalutamide treatment conditions, as a proxy for the whole-body influence of hormones in the mice. However, these data may also be affected by the presence of tumor cells in the spleen (Figure 3G illustrates splenic metastases), as there were variable slight differences across donors and hormone conditions.

### Xenografts in huNOG-EXL mice reveal a suppressed immune profile.

**Figure 4:**
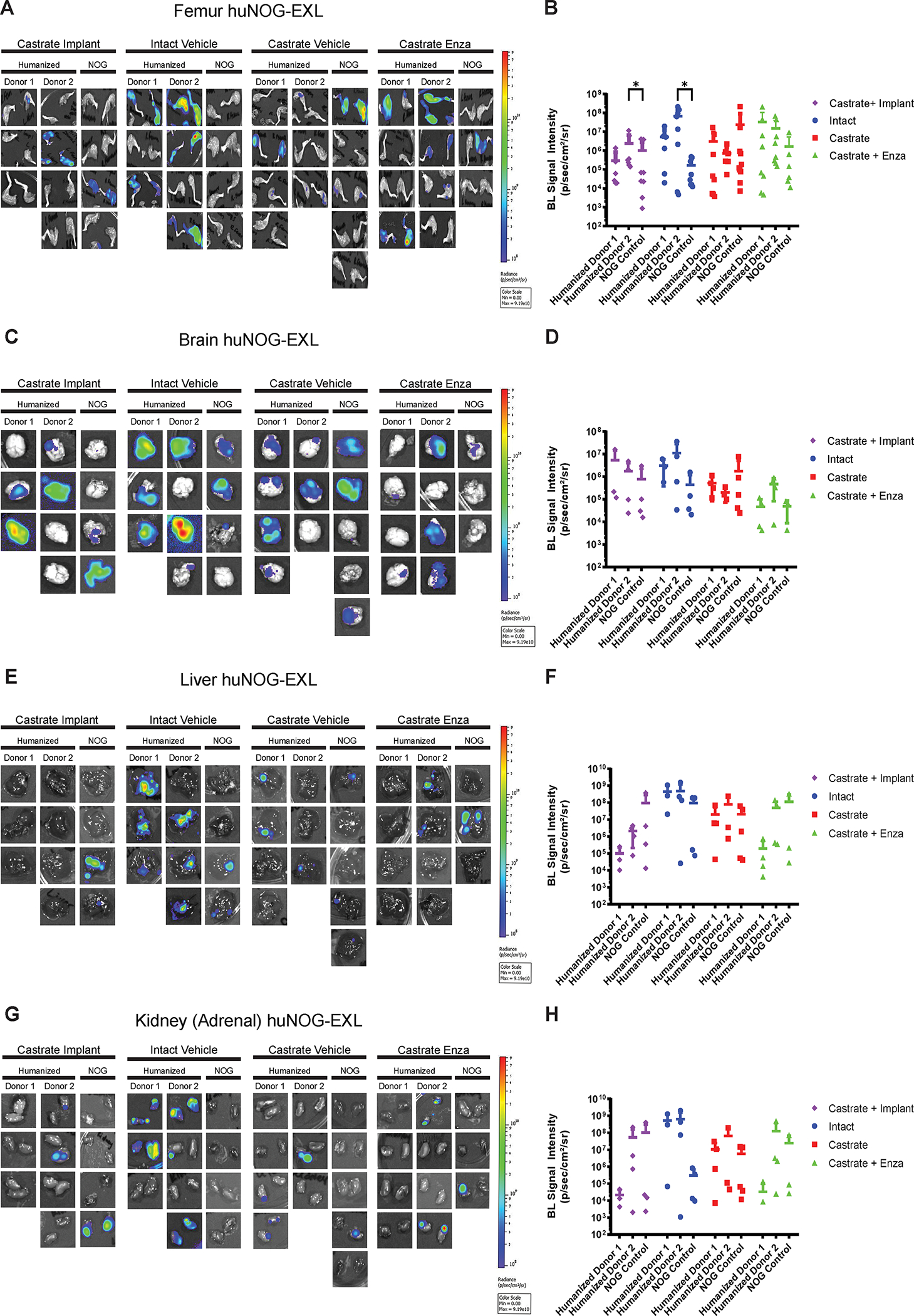
Metastasis by 22Rv1 to additional clinically relevant organs in huNOG-EXL mice. **A,** Bioluminescent images taken of femurs, **B,** Quantified bioluminescence of femur metastasis. **C,** Bioluminescent images taken of mouse brain, **D,** Quantified bioluminescence of brain metastasis. **E,** Bioluminescent images taken of mouse liver, **F,** Quantified bioluminescence of liver metastasis. **G,** Kidney (adrenal gland) bioluminescent images, **H,** Quantified bioluminescence of Kidney (tumor mostly in adrenal gland).

Our results in 22Rv1-engrafted huNOG mice suggest that this mouse model with intact human lymphocytic cells more closely models the patient castration/enzalutamide response than a model using immunocompromised mice; however, immunotherapy responses in prostate cancer patients are low and are often characterized by dense myeloid infiltration (25). We evaluated tumor growth and metastasis in a humanized-NOG mouse system able to maintain not only cells of human lymphocyte lineage, but also human myeloid cells. The huNOG-EXL mice are modified to express human GM-CSF and IL-3 allowing these mice to support human immune cells of myeloid lineage, in addition to lymphocytes. We evaluated subcutaneous tumor growth and measured metastasis in 22Rv1-engrafted huNOG-EXL mice under various levels of androgenic signaling. Mice were divided into four different conditions, listed in descending order of AR-activity outlined in Sedelaar et al. 2013 (26): 1) mice castrated and implanted with testosterone to raise and maintain testosterone levels (530 ± 50 ng/dL) that are physiologically relevant in normal humans (26); 2) mice with intact gonads, thus mimicking hypogonadal or castrate human levels; 3) mice castrated, thus mimicking patients treated with abiraterone (the CYP17A inhibitor (27)); 4) mice castrated and dosed with enzalutamide, thus mimicking androgens depleted and AR-antagonized conditions. Similar to the result in 22Rv1-engrafted huNOG mice with two different HSC donors, primary tumor growth was not affected by either castration or enzalutamide treatment (Figure S8). However, unlike 22Rv1-engrafted huNOG, no difference in metastasis was observed in the huNOG-EXL mice treated with enzalutamide (Figure 4A–H), suggesting a possible role of mature myeloid cells in preventing the enzalutamide effect on metastatic growth seen in huNOG mice. This suggests the possibility of immunosuppressive myeloid cells in tumors of huNOG-EXL mice.

**Figure 5:**
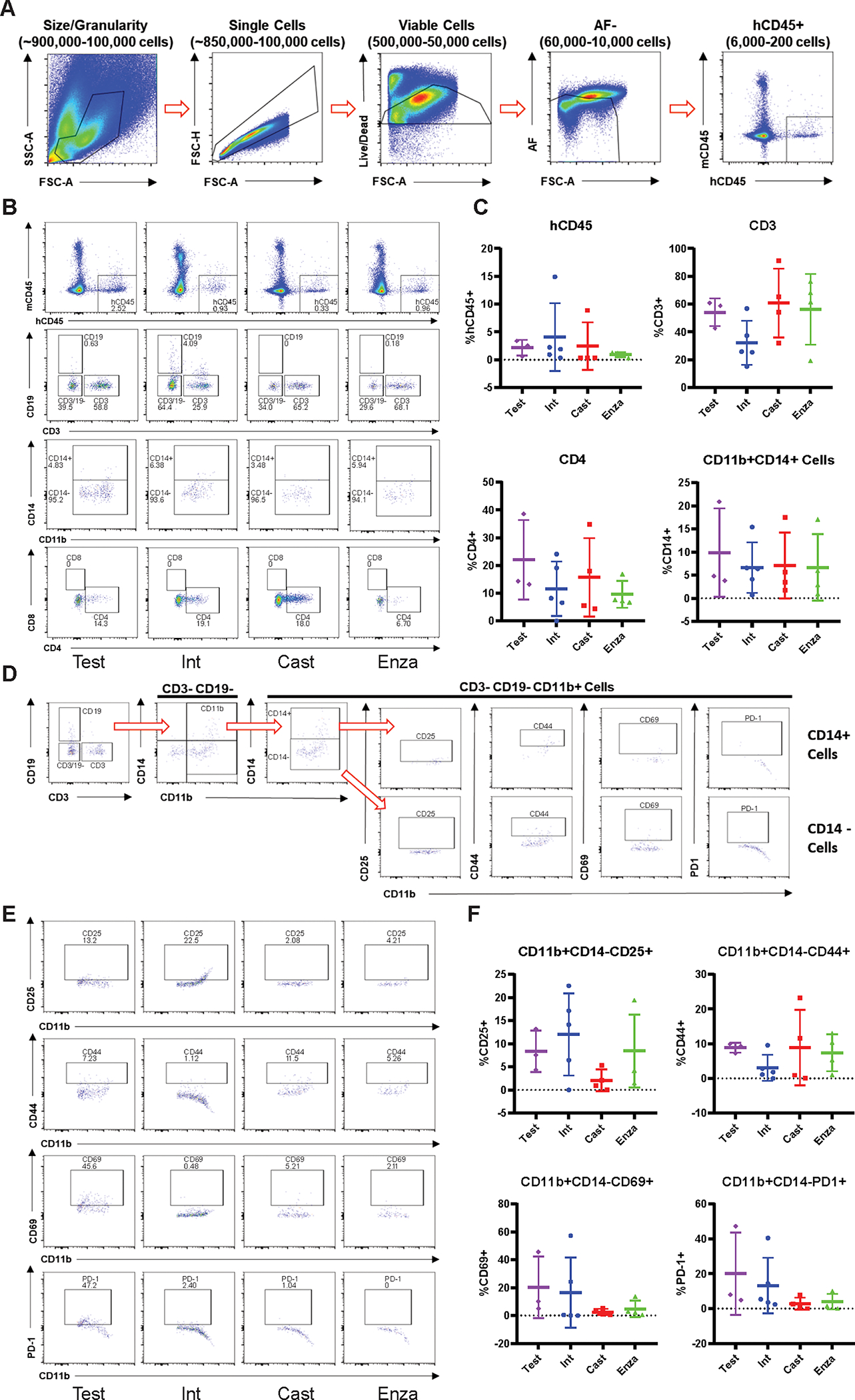
Myeloid-support exhibits immuno-dampened profile in huNOG-EXL 22Rv1 xenograft tumors. **A,** Gating strategy used to determine presence of human CD45+ cells in the NOG-EXL model. **B,** Representative data showing the abundance of various immune cell populations; human leukocytes, CD19+ cells, CD3+ cells and double negative cells, MDSC and activated myeloid cells and helper T-cells (CD4+) and cytotoxic T-cells (CD8+) (top to bottom). **C,** Quantitated data comparing human CD45, CD3, CD4, and CD11b populations in tumors isolated from testosterone implanted vehicle, intact vehicle, castrated vehicle, and castrated enzalutamide treated mice. **D,** Gating strategy for determining the activation state of MDSCs (CD3-CD19- CD11b+ CD14-). **E,** Representative data showing the activation state of the MDSC cells harvested from tumors under different treatment categories through the presence of the surface markers: CD25, CD44, CD69 and PD-1. **F,** Quantitated data showing the activation state of the MDSCs throughout the different treatments.

Our data from TILs (Figure 3) showed that there are activated T-cells in the tumors of 22Rv1-engrafted huNOG mice. We immunologically profiled subcutaneous tumors from 22Rv1-engrafted huNOG-EXL mice using the Cytek Aurora ^®^ system to understand the difference in enzalutamide effect in huNOG compared to huNOG-EXL mice (See details of antibody panel in methods). TILs from 22Rv1-engrafted huNOG-EXL mice tumors were collected for immune profiling from treatment groups with descending levels of androgenic signaling as described for Figure 4 [either castrated/testosterone implant (Test), intact/vehicle (Int), castrated/vehicle (Cast) or castrated/enzalutamide (Enza)]. Analysis of the population of leukocytes (CD45+), T-cells (CD3+), helper T-cells (CD4+) and myeloid cells (CD3- CD19- CD11b+) (Figure 5A) showed no differences between the treatments in the intra-tumoral populations of these immune cells (Figure 5B–C) nor any major differences within the spleens (Figures S9–S11) in the huNOG-EXL model, with similar levels of human leukocyte percentages in the overall tumor. The populations, and activation levels determined by CD25 [the IL-2 receptor, induced upon initial T-cell activation, constitutively expressed in T-regulatory cells (Tregs) (28)], CD44 [upregulated upon activation in effector and memory T-cells (29)], CD69 [a broadly expressed early activation marker in leukocytes implicated in retaining cells in peripheral tissues (30)] and PD1 [programmed cell death-1, a marker of T-cell exhaustion and Treg differentiation (31)] abundance (Figure 5D), of CD14 negative myeloid cells also showed no differences between the treatments in this model (Figure 5E–F). The activation level was low, suggesting the huNOG-EXL represents an immune “cold” model similar to what is seen in the majority of prostate cancer patients (32,33). The population of CD14+ myeloid cells observed was too small to determine their level of activation (Figure 5). In fact, it is interesting to note that for all treatments, the majority of myeloid cells were CD14-negative, which is indicative of immature myeloid derived suppressor cells (MDSCs) (34). We identified very few tumor-infiltrating B-cells (CD19 positive) and Natural Killer (NK) cells (CD56+, CD16+) across hormone conditions, despite high levels in the spleens of these mice (Figure S11 E–G).

**Figure 6:**
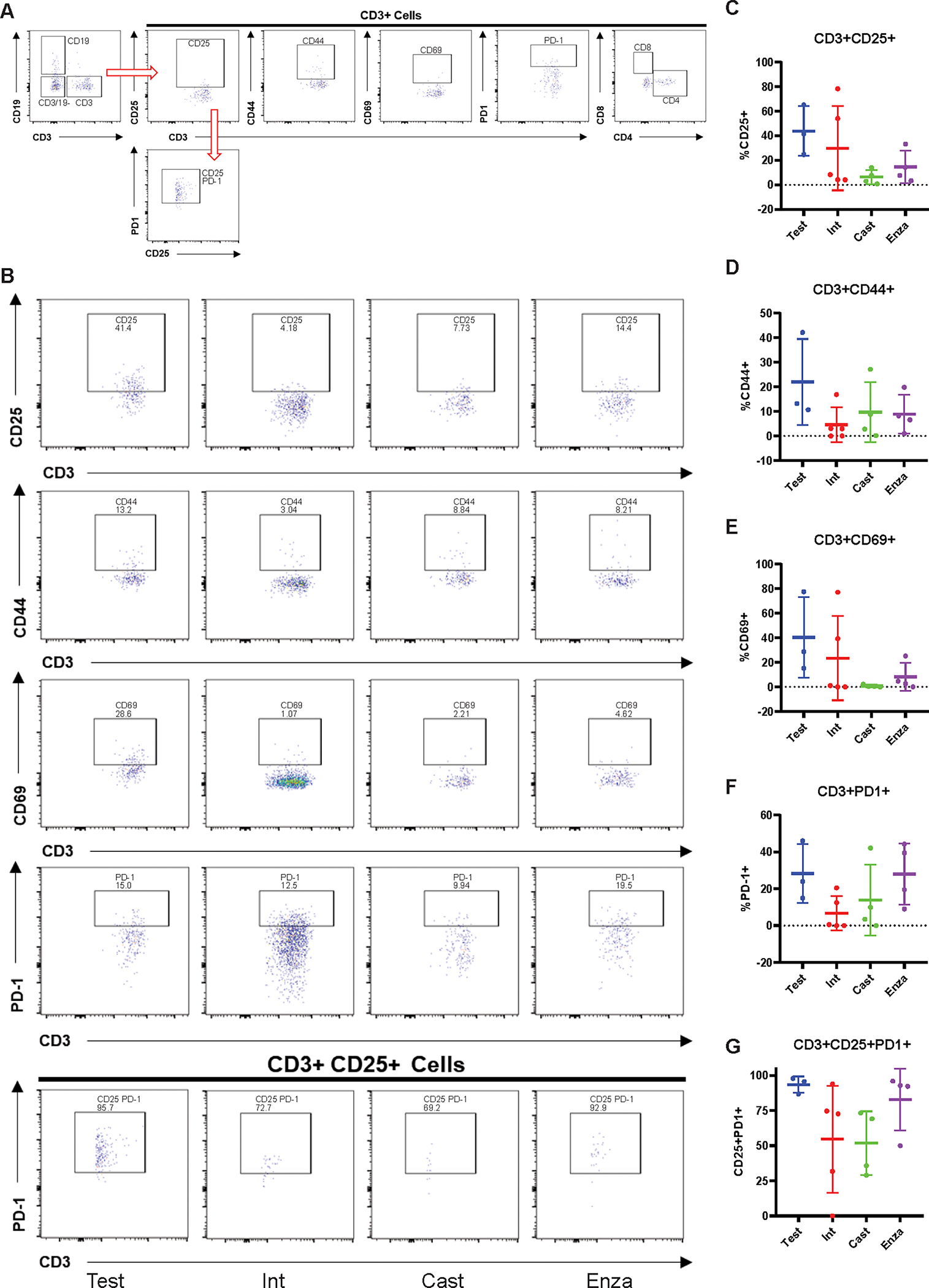
Immune-dampened profile of T-cells in huNOG-EXL tumors. **A,** Gating strategy for determining the activation state of T-cells and regulatory-like T-cells. **B,** Representative data showing the activation state of the CD3+ cells harvested from tumors under different treatment categories through the presence of the surface markers: CD25, CD44, CD69 and PD-1 and regulatory-like cells (CD3+CD25+PD-1+). **C,** Quantitation showing the expression of CD25, **D,** CD44, **E,** CD69, **F,** PD-1, **G,** regulatory-like T-cells.

We also profiled T-cells from 22Rv1-engrafted huNOG-EXL mice tumors for activation markers and to determine the population of Tregs (Figure 6A). Although no differences were observed between treatment groups (Figure 6B–6G), for the CD3+ CD25+ cells (Figure 6C), the majority were PD1+ (Figure 6F) which is indicative of the regulatory T-cell (together in Figure 6G) phenotype found in the bone-microenvironment of prostate cancer patients (16). We confirmed these findings through multiplexed immunofluorescence (IF) and immunohistochemical (IHC) staining of tumors for CD3, CD8 and Granzyme B expression (IF-Figure S11) and AR and PD-1 expression (IHC-Figure S12). We find few CD3+ T-cells within these tumors, even fewer CD8+ cells, with even fewer of them expressing the activation/effector molecule Granzyme B (35). Consistent with the flow cytometry results, we find few differences across all groups for their expression (Figure S12), with some slight elevation detected in one liver metastasis, and no significant differences in AR and PD-1 staining across the treatment groups (Figure S13), with PD-1+ cells detected in all tumors. This result, together with the data on MDSCs (Figure 5) suggests a suppressed immune profile (“cold”) in the huNOG-EXL system, in contrast to the activated immune profile (“hot”) seen in the huNOG model (Figure 3).

### VCaP Xenograft Tumors respond to Immunotherapy and Enzalutamide in the huNOG Model.

**Figure 7:**
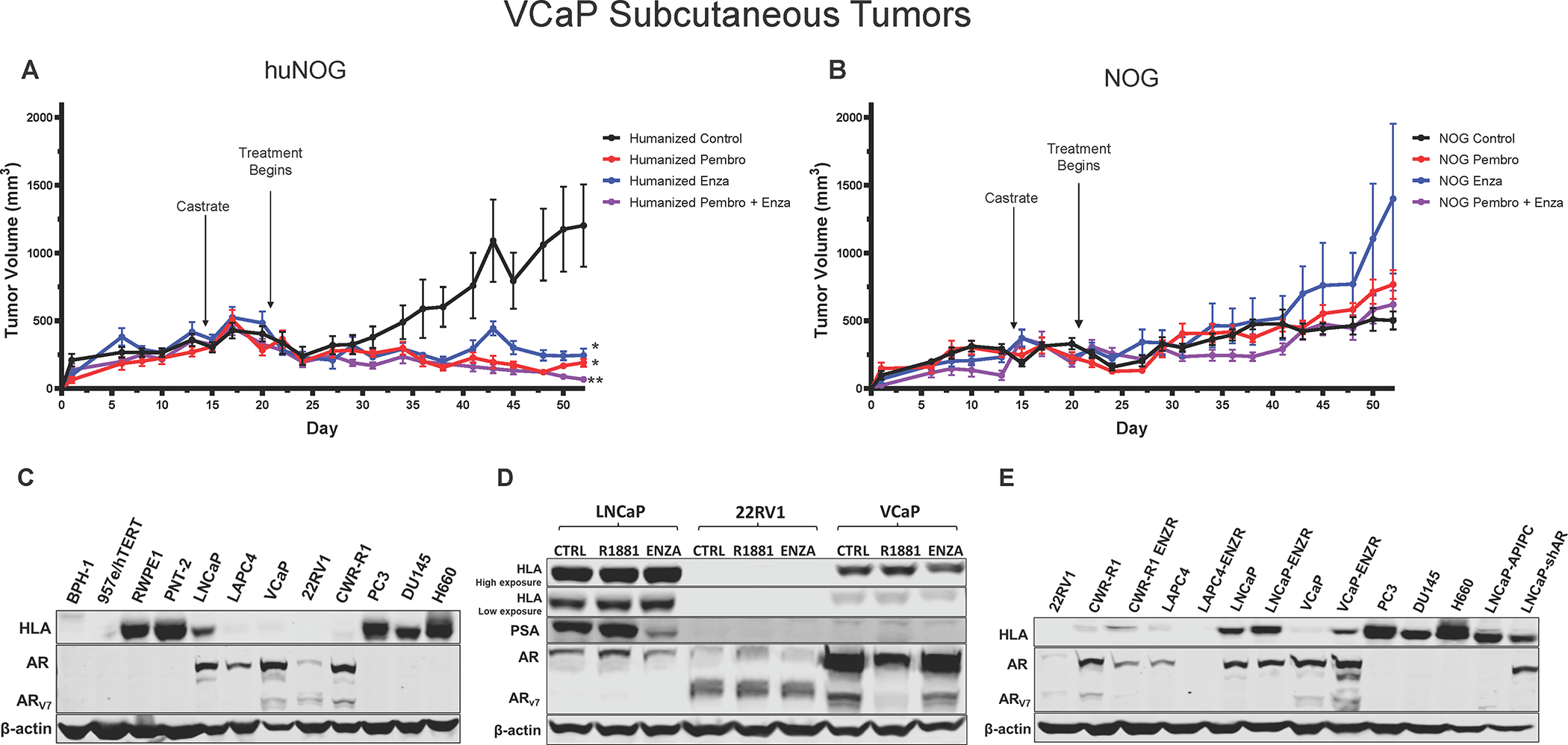
Response of VCaP subcutaneous tumors to anti-PD1 immunotherapy and enzalutamide. **A,** huNOG primary tumor growth response and **B,** NOG primary growth response to pembrolizumab, enzalutamide and combination therapy. * Represents p>0.05 when compared to vehicle control, ** represents p>0.05 when compared to all other conditions. **C,** HLA (HLA- A, -B, -C) expression of a panel of benign prostate (BPH-1, 957e/hTERT, RWPE1, PNT-2), AR-positive prostate cancer (LNCaP, LAPC-4, VCaP, 22Rv1, CWR-R1) and AR-negative (PC3, DU145 and NCI-H660) cancer cell lines determined by western blotting (representative blots show, experiment replicated three times). Corresponding AR blot and AR variant (AR-V7) detection. **D,** LNCaP, 22Rv1 and VCaP HLA response the AR agonist, R1881 (1 nM), AR-antagonist enzalutamide (ENZA - 10 μM) and vehicle control (CTRL) treatment for 24 hours. PSA is a canonical AR-target gene as a readout of AR modulation. **E,** Comparison of HLA expression between enzalutamide naïve and resistant cell lines (ENZR).

To test another xenograft model in humanized mice, we relied on the VCaP castration-resistant-xenograft model, which mimics the natural history of prostate cancer, with short-term responses to both castration and AR-antagonism (19). We assayed primary tumor growth using a VCaP castration resistant model in huNOG mice to evaluate the model in a more enzalutamide sensitive setting, determine if we could enhance the effects of immune activation by treatment with a checkpoint inhibitor in the form of the anti-PD1 pembrolizumab (pembro). In this model VCaP tumor–bearing mice were castrated when the tumors were approximately 200 mm^3^ in size, and once the tumor grew back, animals were randomized and treated with enzalutamide and/or the anti-PD-1 antibody pembro, or with vehicle controls. VCaP xenograft tumors treated with enza and pembro work well as monotherapies, and slightly better in combination, completely eliminating tumors. (Figure 7A). NOG mice were not responsive to either of these treatments (Figure 7B). This result demonstrates that this model is responsive to immune checkpoint inhibition and works synergistically with AR targeted therapy.

Finally, to provide some insight of the mechanism of the anti-tumor effects seen in the huNOG mice, we assayed prostate cancer cells for HLA expression (Figure 7C) and to identify Androgen-control of HLA (-A, -B, -C) expression. 22Rv1 cells display no expression of HLA molecules, regardless of AR modulation (Figure 7 C, D, E), however, VCaP cells illustrated increased HLA expression in the enzalutamide-resistance state (Figure 7D), and slight decreases with short-term treatment (Figure 7E). Other AR-positive prostate cancer cell lines generally display decreases in HLA expression when compared to immortalized benign controls (Figure 7C), and cells that maintain AR expression after enza-resistance show increased HLA expression, as evidenced by previous reports (2,33,36,37). Taken together, these data suggest that much of this response is likely governed by the T-cell activation seen with enza-treatment (38), as well as some potential cancer cell autonomous effects, all of which are topics for future investigation.

## Discussion

To the best of our knowledge, these results illustrate the first model of human PCa that metastasizes to clinically relevant locations, has an intact human immune system, and responds appropriately to the standard-of-care hormonal therapies. We found that humanizing tumor-immune interactions improved modeling of metastatic PCa and provides a model more suitable to evaluate hormonal and immuno- therapies. These huNOG mice provide a model that not only has an intact human immune system, but also shows metastases to relevant secondary sites and models the effect anti-androgen treatment has on metastasis. In 22Rv1 xenografts, subcutaneous tumor size was not significantly altered across hormone conditions; however, metastasis to secondary sites differed in castrate huNOG mice treated with enza versus background-matched immunocompromised mice. VCaP xenograft tumors showed decreases in growth with enza and anti-Programed-Death-1 treatments in huNOG mice, with combination eliminating tumors, and no effect was seen with any treatment in NOG mice. Enza responses in huNOG and NOG mice were distinct and associated with increased T-cells within tumors of enza-treated huNOG mice, and increased T-cell activation. In huNOG-EXL mice, which support human myeloid development, there was a strong population of immunosuppressive regulatory T-cells and MDSCs, and enza treatment showed no difference in metastasis.

The 22Rv1-engrafted huNOG mouse models human PCa in that it metastasizes to clinically relevant locations. Metastasis was observed in bone (femur and humerus), lymph nodes, liver, brain, spleen, lungs, and kidneys (adrenal) in 22Rv1-engrafted huNOG as well as in the immunocompromised NOG mice. However, reduction of metastasis by enzalutamide was only observed in the huNOG mice. In the immunocompromised NOG mice, enzalutamide treatment did not decrease metastasis, but instead showed a paradoxical increase in metastases. The decreased metastasis with enzalutamide treatment in the huNOG mice is consistent with the clinical situation where Hussain *et al*. found that enzalutamide treatment of patients with non-metastatic CRPC had a 71% lower risk of metastasis (20). In this way, the xenograft model in huNOG mice has the advantage in that it responds appropriately to standard-of-care hormonal therapies.

An important feature of this xenograft model in huNOG mice is the presence of tumor-immune interactions. The huNOG mice maintain cells of the human immune system, including functioning human T-cells. Taken together, the results suggest that the effect of enzalutamide to decrease metastasis involves an interaction with the immune system. This hypothesis is supported by the presence of increased infiltration of activated (INFγ+) T-cells into the tumors from the huNOG mice treated with enzalutamide, but surprisingly, no differences in TNFα were observed (Figure S6). As androgen signaling has been shown to be immunosuppressive (38,39), AR-antagonism might relieve an inhibitory signal and activate immune surveillance, as well as promote T-cell tumor infiltration. These results suggest that a huNOG xenograft model exhibits an activated “hot” immune profile from an immune perspective, and this may be important in the ability of the model to replicate the action of enzalutamide to decrease metastasis as seen in the clinical situation.

Interestingly, enzalutamide’s ability to decrease metastasis was not observed in the huNOG-EXL model. The huNOG-EXL mice maintain human immune cells of both a myeloid and lymphoid lineage. The TILs from tumors from enzalutamide treated huNOG-EXL mice did not show the increased infiltration of activated T-cells that was observed with the huNOG mice, and the presence of MDSC-like cells and Tregs suggests a suppressed “cold” immune profile in the huNOG-EXL system. This immunosuppressed profile may account for the lack of effect of enzalutamide on metastasis in this system. Enzalutamide itself has been shown to promote immunosuppressive effects in myeloid populations through a non-AR dependent manner (40).

The huNOG mouse xenograft model can be used for other PCa tumor cell lines, besides 22Rv1. Using a VCaP castration resistant model in huNOG mice, we demonstrated the usefulness of a huNOG mouse xenograft to evaluate immunotherapies, either as monotherapies or in combination with a hormone treatment such as enzalutamide. VCaP xenograft tumors were responsive to the anti-PD1 pembrolizumab as a monotherapy, and in combination with enzalutamide, worked synergically (Figure 7A). Tumors in immunocompromised NOG mice were not responsive to either of these treatments (Figure 7B).

Future work will be focused dissecting the mechanism of how AR and its signaling exhibits the cell-specific effects within prostate tumors, and how this affects interactions between cancer cells and immune cells in the tumor microenvironment to produce differential responses to AR targeted therapies. One important question is the role of PD-1 expression or the presence of Tregs in the huNOG model. Unfortunately, the low overall abundance of T-cells compared to 22Rv1 tumor cells in the huNOG subcutaneous xenografts limited our ability to reliably assay for Tregs by flow cytometry and histological assessments were similarly unreliable. However, this interesting concept should be explored further due to the dominant role that T-cells play in this model. Additionally, we observed that enzalutamide resistant cells upregulate HLA, here (Figure 7E) and as previously reported (2), but others have also illustrated a connection between chronic enzalutamide treatment and upregulation of immune-regulatory proteins that produce an immunosuppressed microenvironment (41). Our models presented here have the potential to aid further work which is necessary to tease apart the timing and phasing of AR inhibition to produce an optimal immune response.

The huNOG mice illustrate decrease metastatic burden with enzalutamide, even with a resistant cell line, and may even show stronger effects in the bone versus visceral organs, which is also observed clinically (42). However, while this better represents what is observed clinically, the huNOG-EXL model better mirrors a typical “cold” prostate immune microenvironment. This is an interesting discrepancy and may have to do with the sites of the primary tumors verses metastases or even how the AR-targeted therapies change HLA expression. One potential future approach to expand on the results of this study is to assess this model using an orthotopic prostate injection rather than a subcutaneous injection of the prostate tumor cells. This would be even more relevant in huNOG-EXL xenografts, given that xenograft tumor site shows differential effects to myeloid and immune checkpoint responses in other tumor types (43), and could provide an approach with a more relevant immune microenvironment. Future studies will also focus on the role of AR in these models, in both the stroma, the cell-autonomous signaling, and tumor-stroma interactions, even in AR-negative prostate cancer cells, which seem to express higher levels of HLA when compared to AR-positive disease (Figure 7).

Excitingly, for a subset of patients, use of biphasic androgen therapy has produced responses to immune checkpoint inhibition (44), which we can also model in our huNOG systems. Finally, we illustrate the use of this model across several cancer cell line derived xenografts; however, we hope to utilize mice with humanized immune systems to model the diverse clinical responses seen with AR- and immune-checkpoint inhibition in other tumor models. Particularly those from patient derived xenografts and organoids, such as CDK12 mutated tumors – some of which are responsive to checkpoint inhibition (45) – and to model clinical resistance to different therapies and disease states that otherwise has been difficult to do with standard xenografts or GEM models.

## Supplementary Material

1

2

3

4

5

6

7

8

9

10

11

12

13

14

## Implications:

To our knowledge, results illustrate the first model of human PCa that has an intact human immune system, metastasizes to clinically relevant locations, responds appropriately to standard-of-care hormonal therapies, and can model both an immunosuppressive and checkpoint-inhibition responsive immune microenvironment.
