# Supplementary figures and images for: Clinically relevant humanized mouse models of metastatic prostate cancer facilitate therapeutic evaluation
Source: Mol Cancer Res. Author manuscript; Available in PMC 2024 Sep 5. (PMC11372372; doi:10.1158/1541-7786.MCR-23-0904)

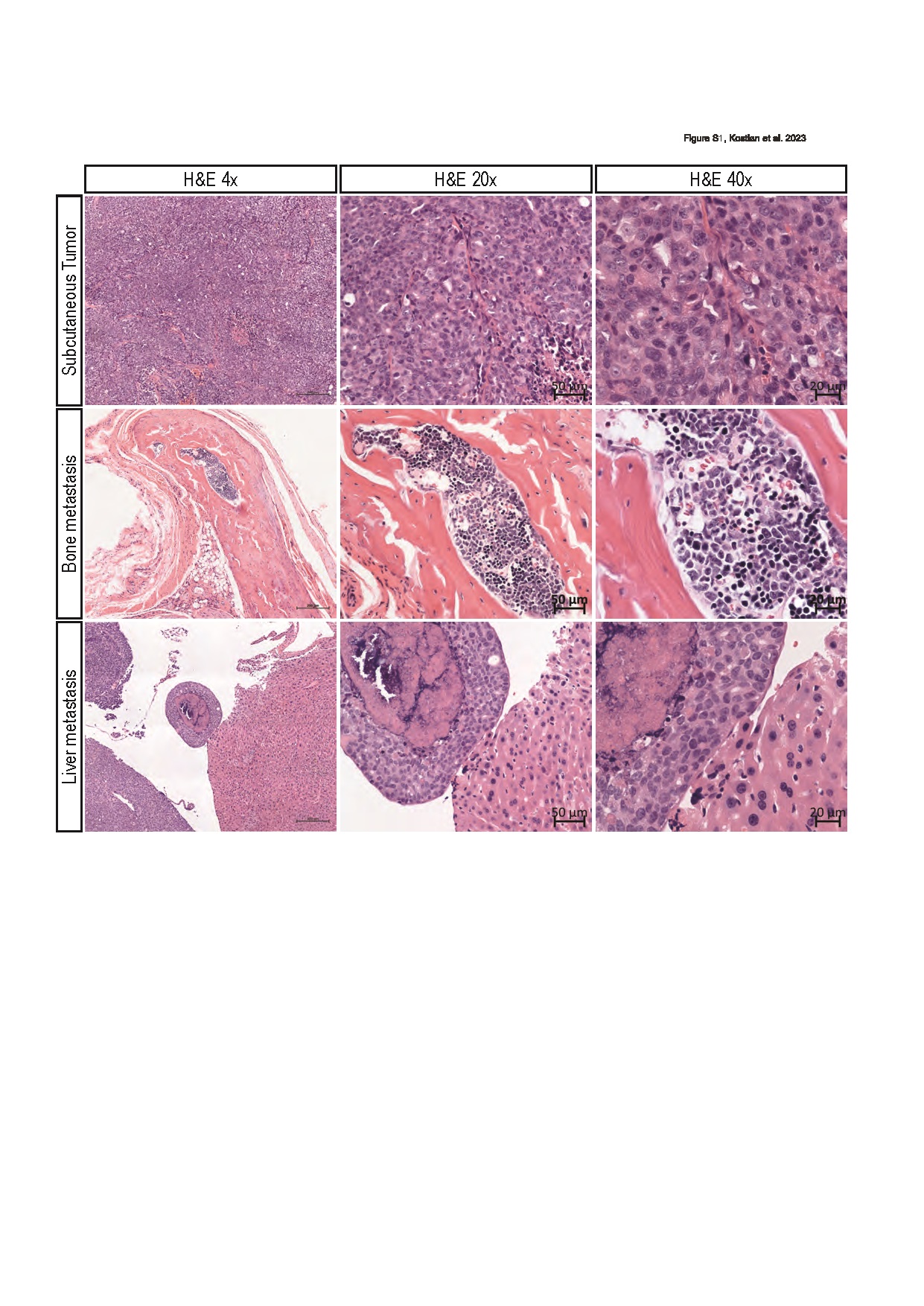

Supplement: 1 [file NIHMS2000254-supplement-1.jpg]

A

Humerus huNOG

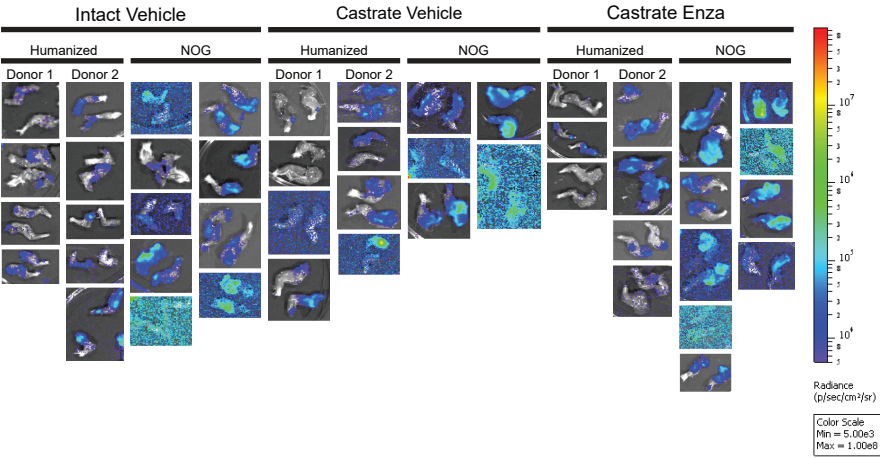

Figure S2, Kostlan et al. 2023

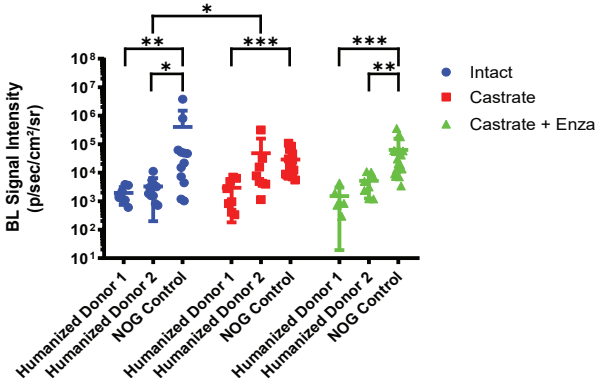

B

Humerus huNOG-EXL

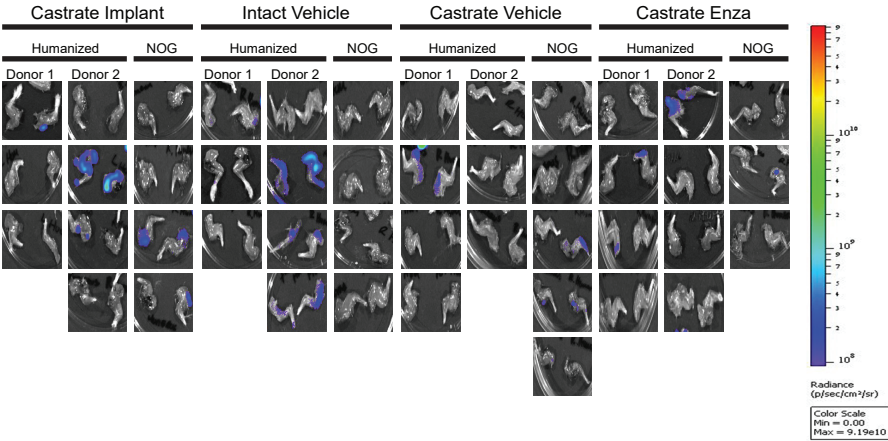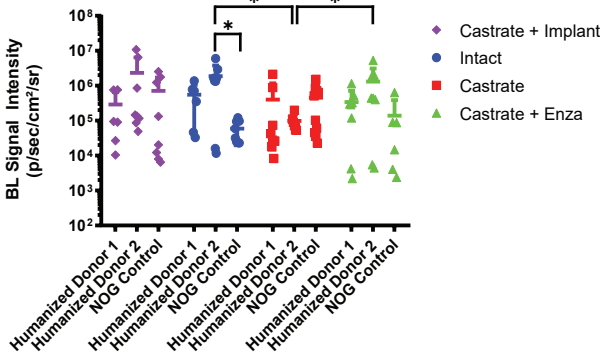

Supplement: 2 [file NIHMS2000254-supplement-2.pdf]

A

## Skull huNOG

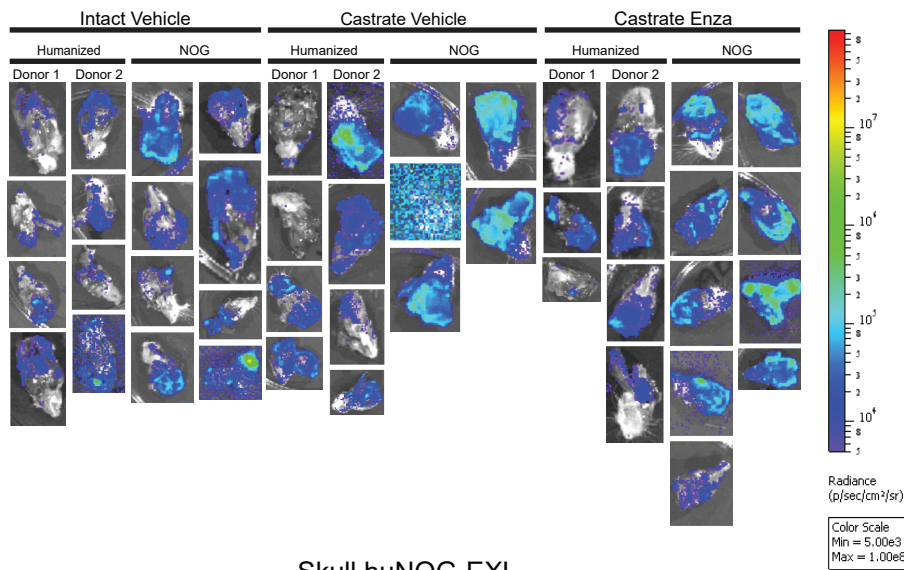

B

## Skull huNOG-EXL

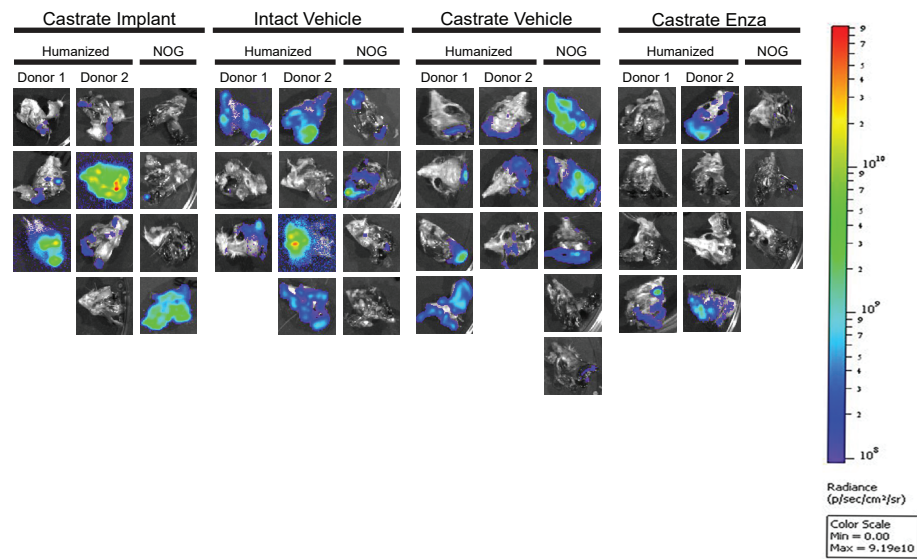

Supplement: 3 [file NIHMS2000254-supplement-3.pdf]

A

## Spleen huNOG

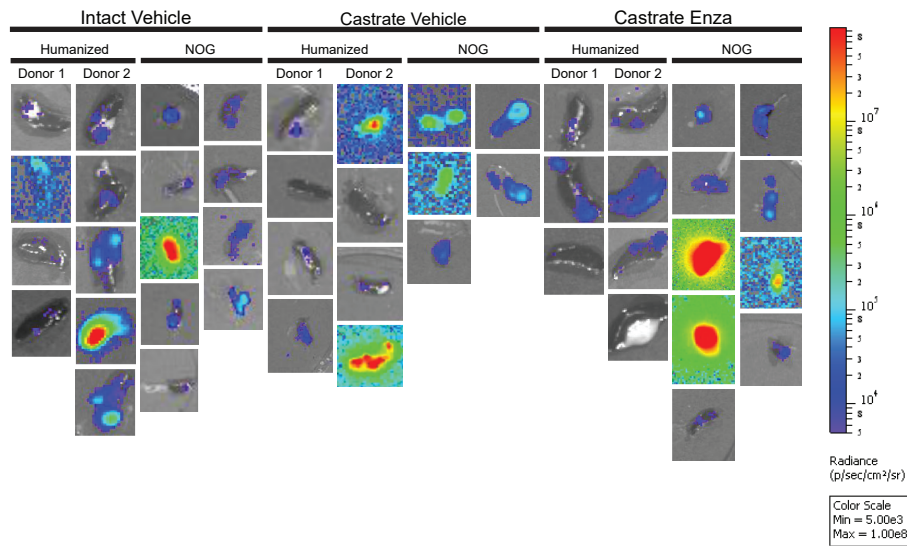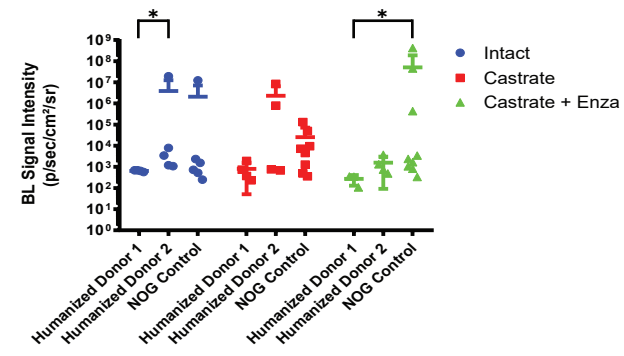

B

## Spleen huNOG-EXL

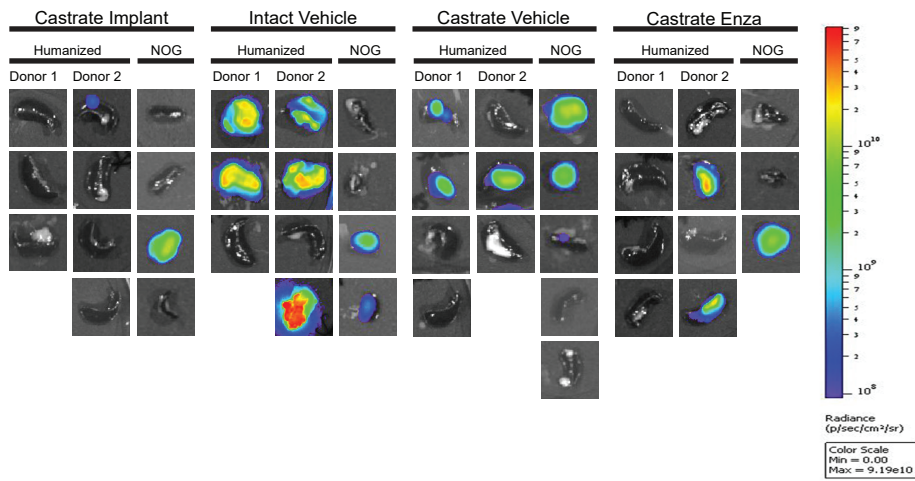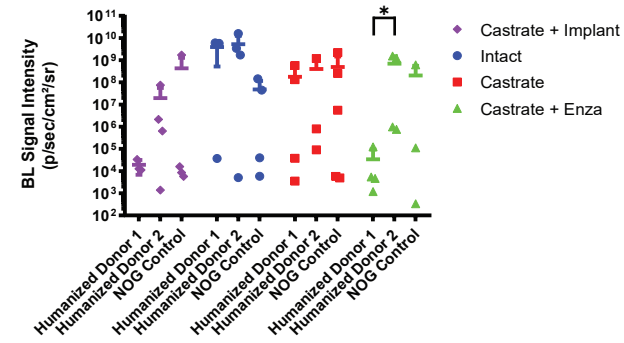

Supplement: 4 [file NIHMS2000254-supplement-4.pdf]

A

## Lung huNOG

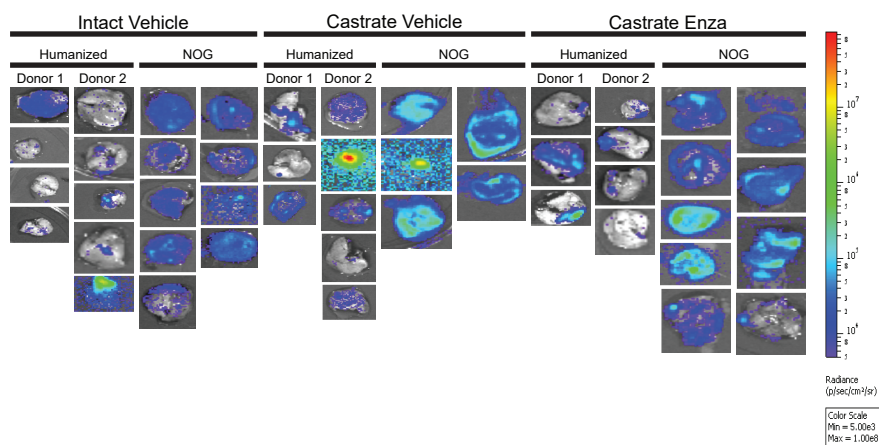

Figure S5, Kostlan et al. 2023

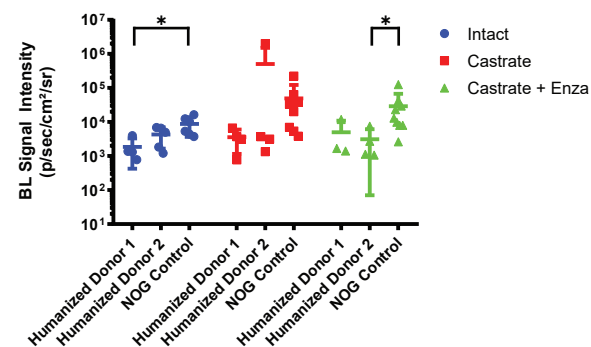

B

## Lung huNOG-EXL

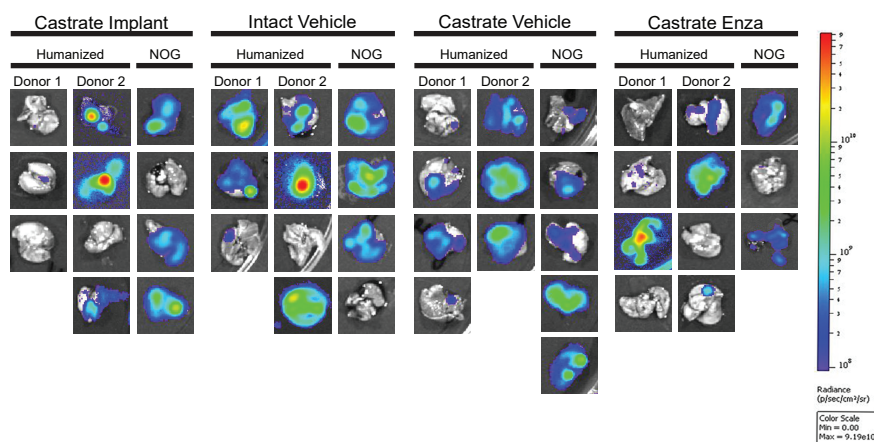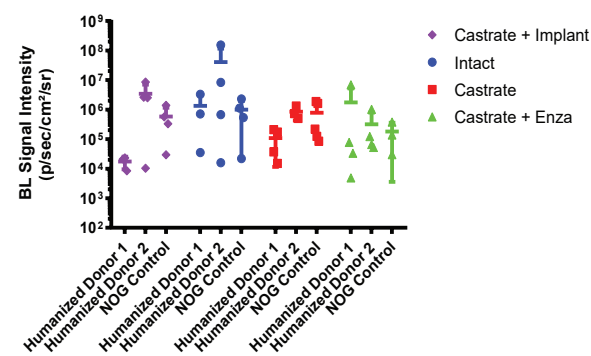

Supplement: 5 [file NIHMS2000254-supplement-5.pdf]

A

Heart huNOG

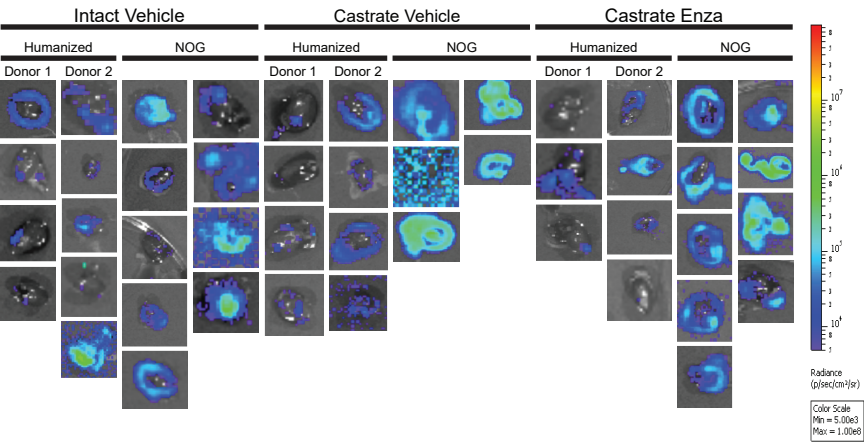

Figure S6, Kostlan et al. 2023

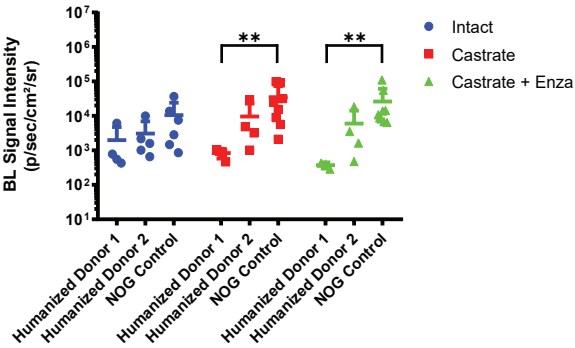

B

Heart huNOG-EXL

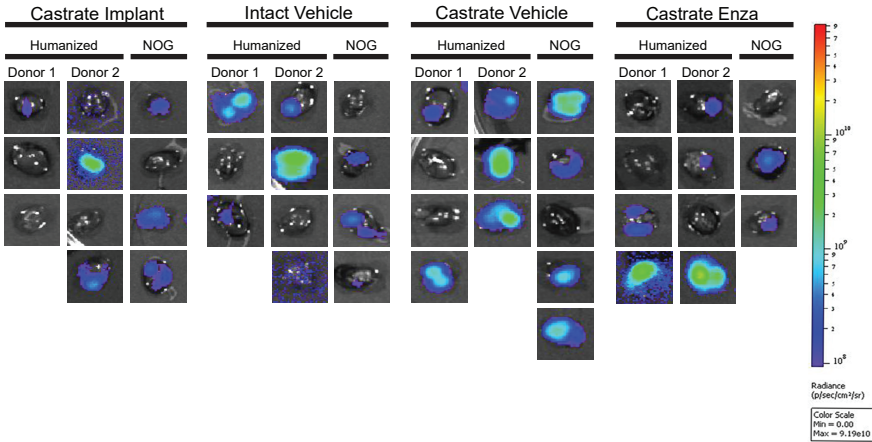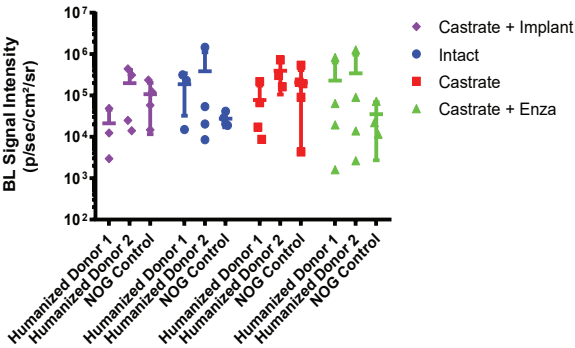

Supplement: 6 [file NIHMS2000254-supplement-6.pdf]

Figure S7, Kostlan et al. 2023

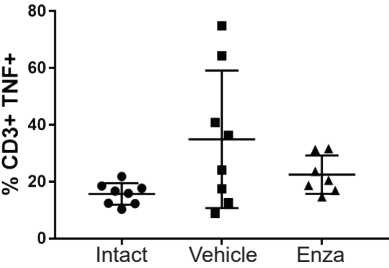

Supplement: 7 [file NIHMS2000254-supplement-7.pdf]

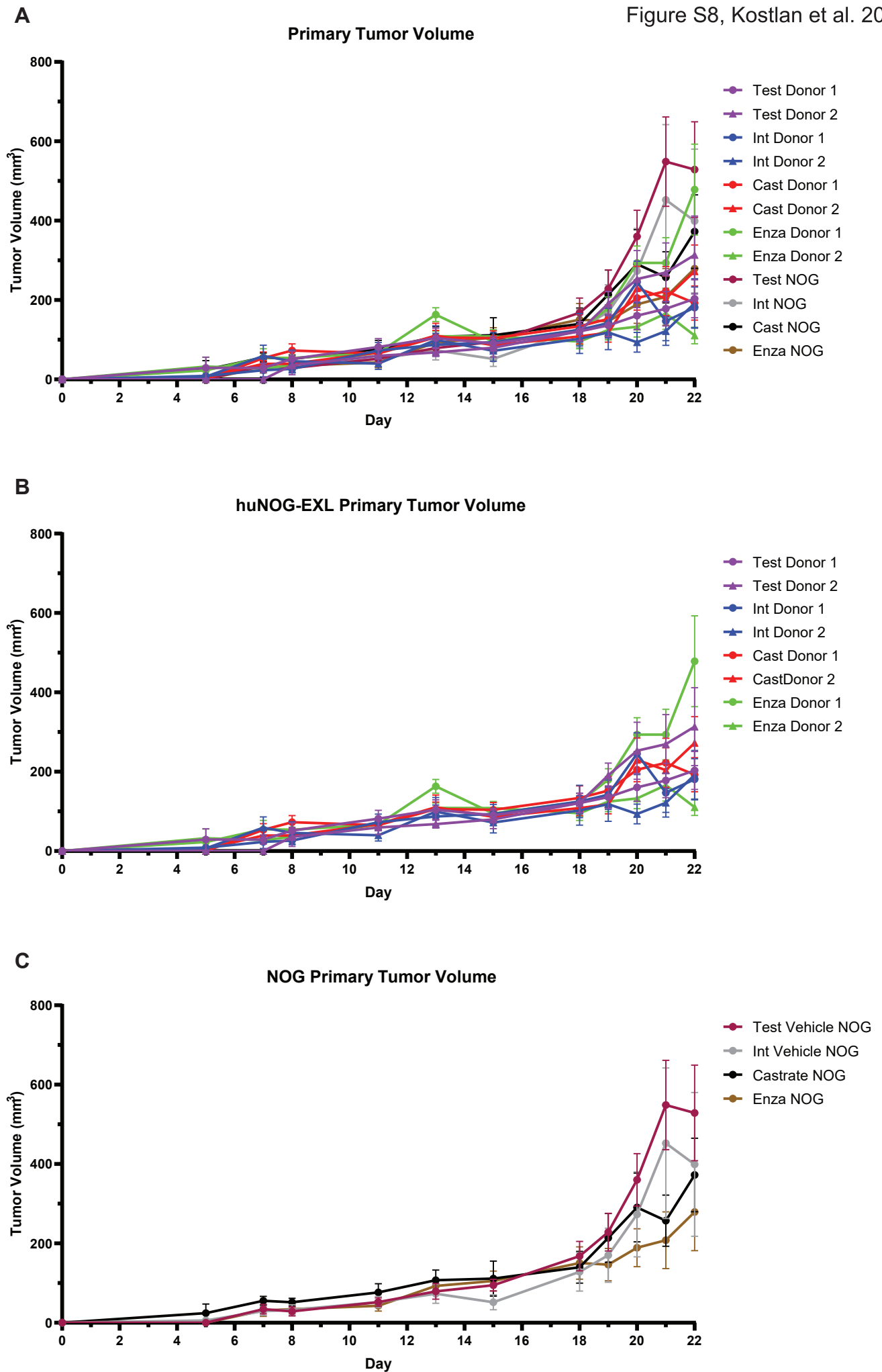

Supplement: 8 [file NIHMS2000254-supplement-8.pdf]

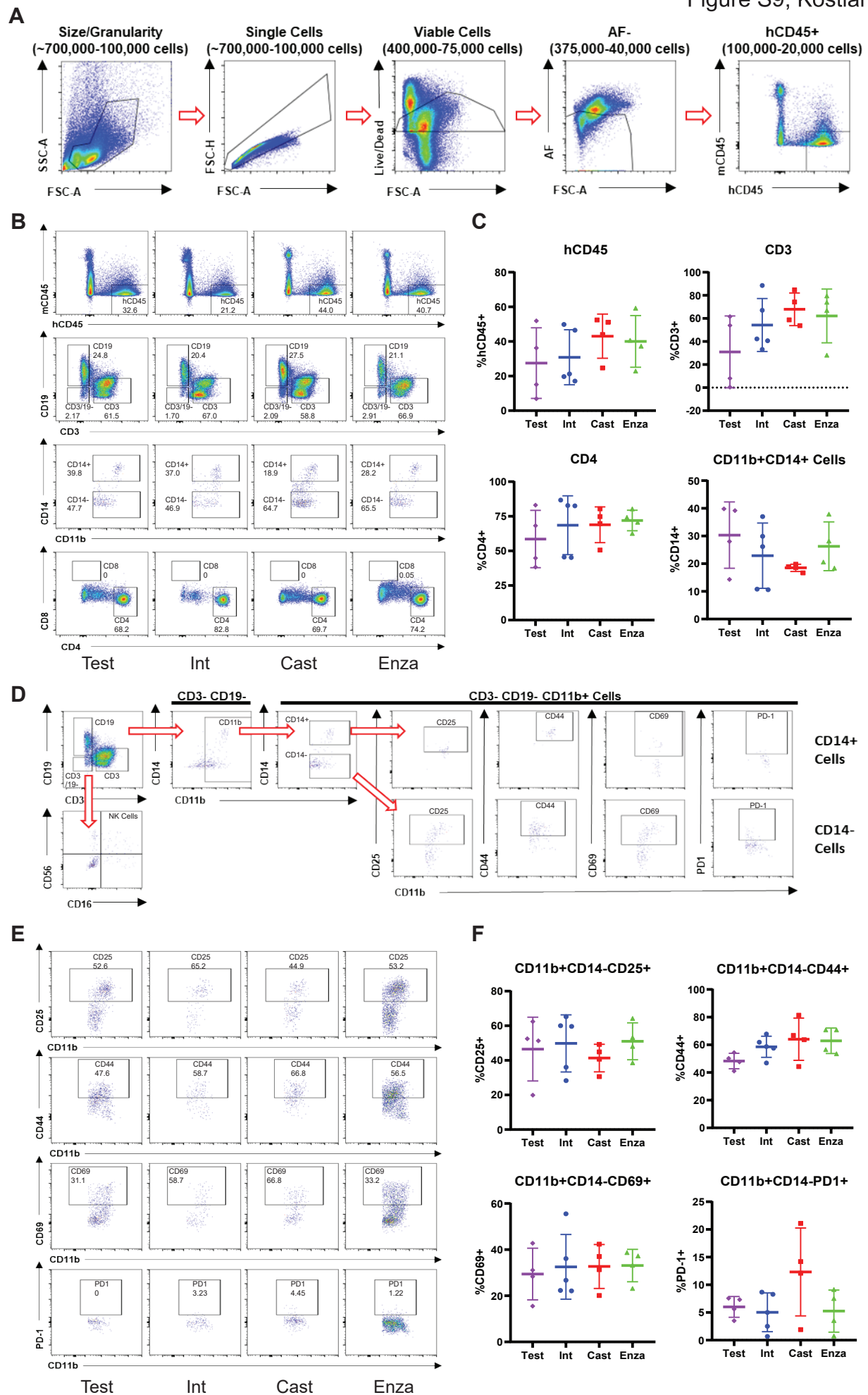

Supplement: 9 [file NIHMS2000254-supplement-9.pdf]

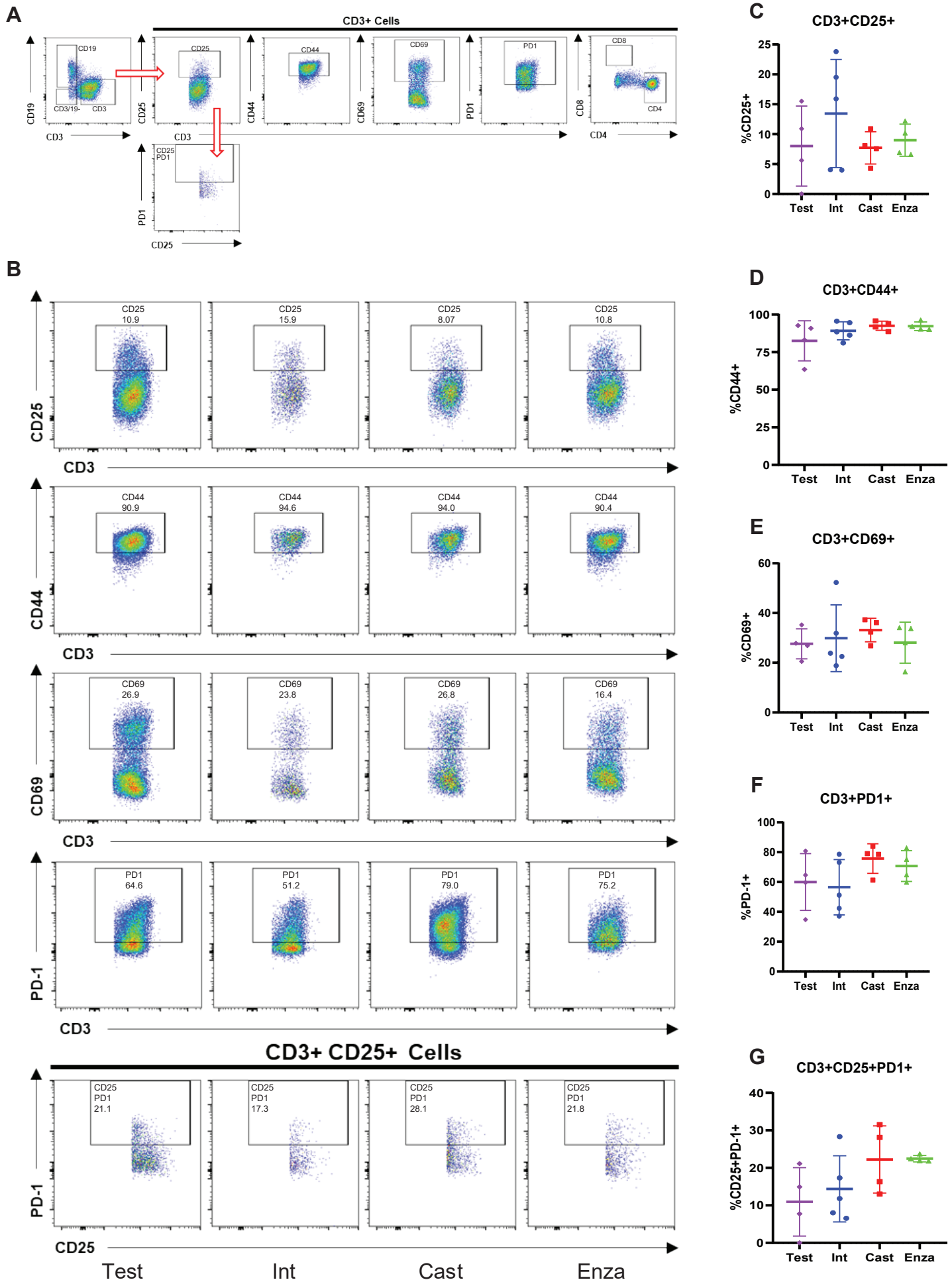

Supplement: 10 [file NIHMS2000254-supplement-10.pdf]

A

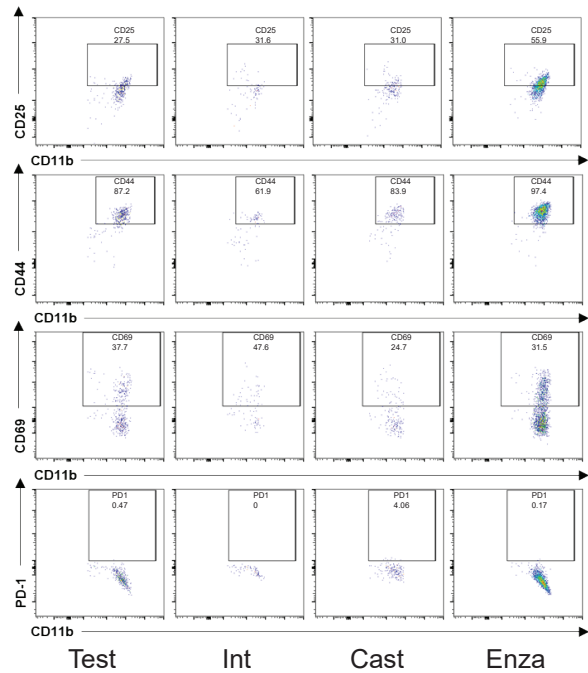

B

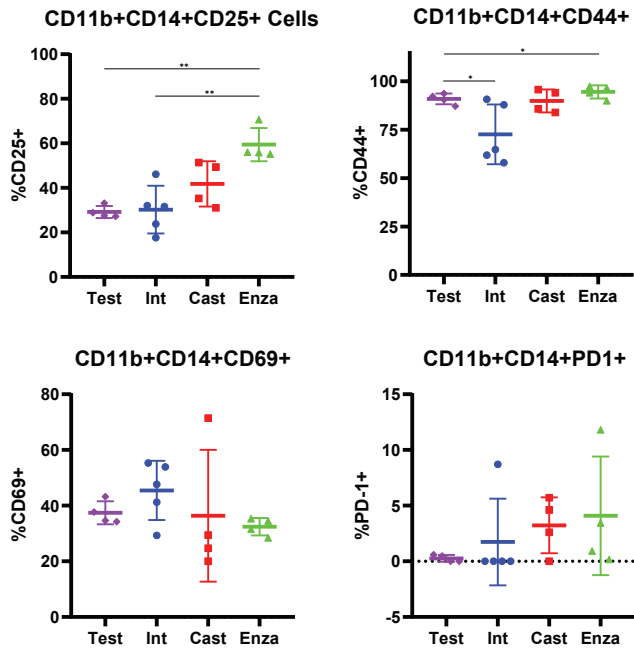

C

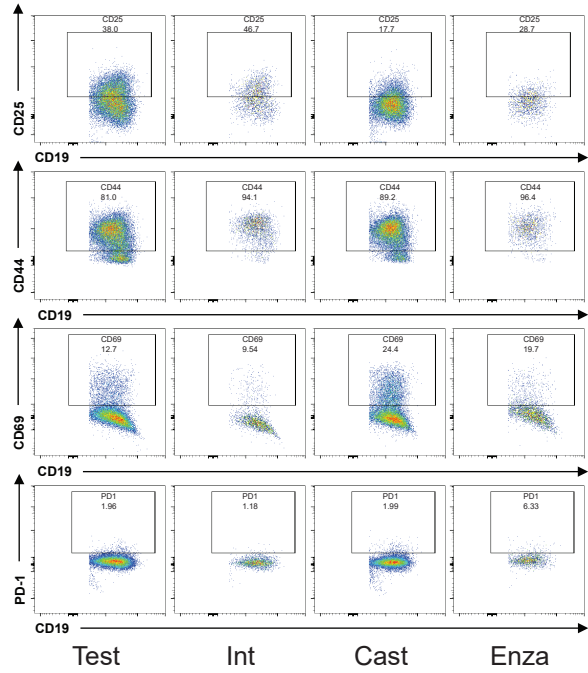

D

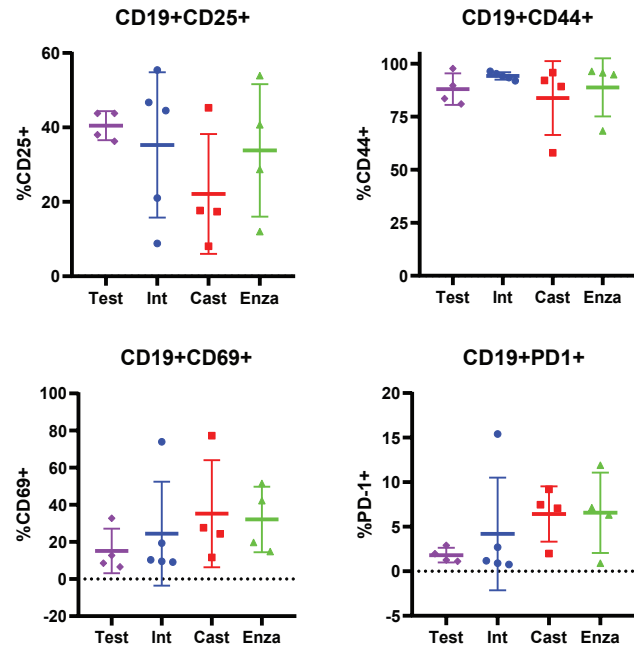

E

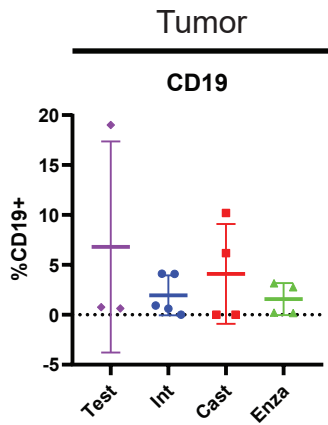

F

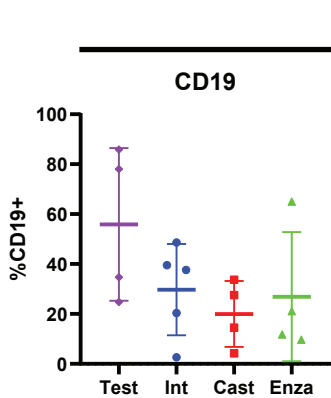

G

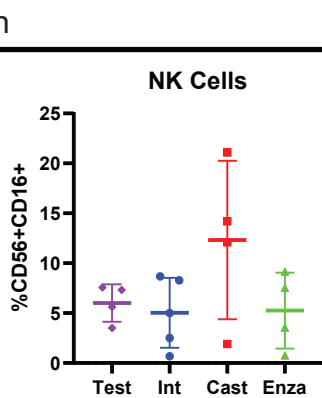

Supplement: 11 [file NIHMS2000254-supplement-11.pdf]

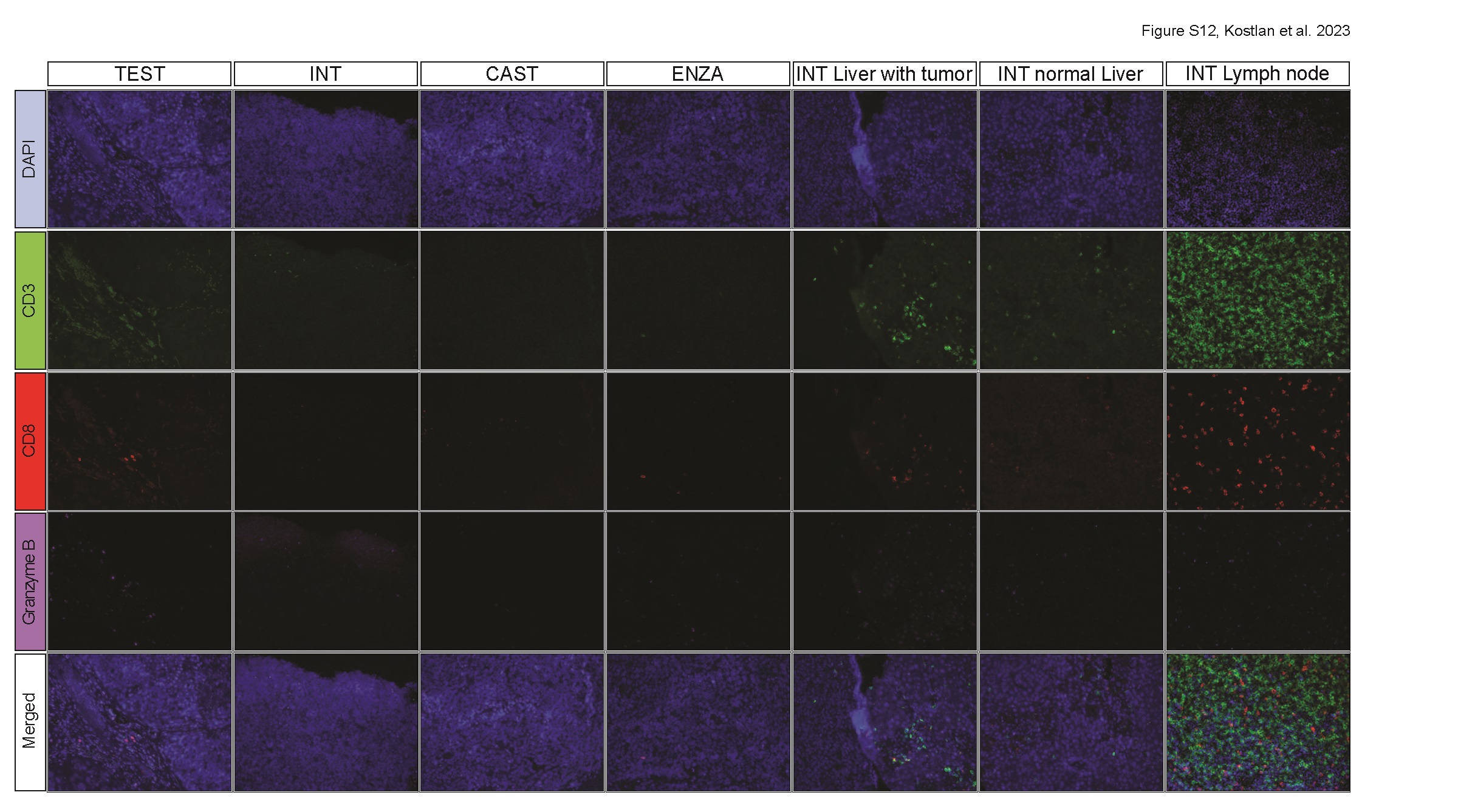

Supplement: 12 [file NIHMS2000254-supplement-12.jpg]

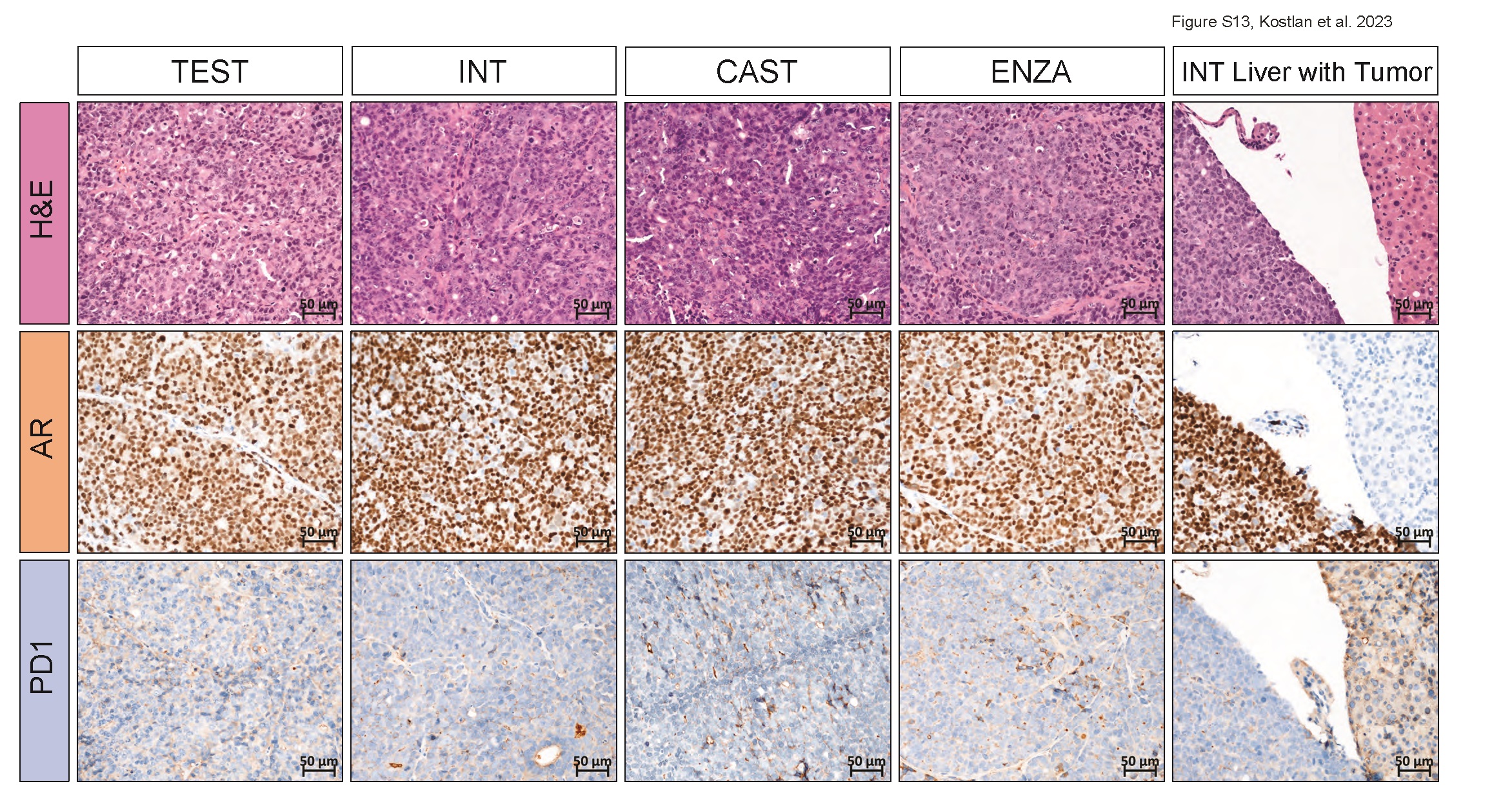

Supplement: 13 [file NIHMS2000254-supplement-13.jpg]
