# Supplementary material for: Clinically relevant humanized mouse models of metastatic prostate cancer facilitate therapeutic evaluation
Source: Mol Cancer Res. Author manuscript; Available in PMC 2024 Sep 5. (PMC11372372; doi:10.1158/1541-7786.MCR-23-0904)
Supplement: 14 [file NIHMS2000254-supplement-14.docx]

Supplementary Figure Legends:

**Figure S1: Representative histology of subcutaneous tumor, bone and liver metastasis of 22Rv1 cells in huNOG mice.** Panels showing representative images at 4x (left column), 20x (middle column) and 40x (right column) magnification of subcutaneous (top row) tumors, and identified bone (femur, middle row) and liver (bottom row) metastases stained by H&E.

**Figure S2: Metastasis by 22Rv1 to the humerus in huNOG and huNOG-EXL mice. A,** Bioluminescent images taken of huNOG humeri, **B,** Quantified bioluminescence of huNOG humerus metastasis. **C,** Bioluminescent images taken of huNOG-EXL humeri, **D,** Quantified bioluminescence of huNOG humerus metastasis.

**Figure S3: Metastasis by 22Rv1 to the skull in huNOG and huNOG-EXL mice. A,** Bioluminescent images taken of huNOG skulls, **B,** Quantified bioluminescence of huNOG skull metastasis. **C,** Bioluminescent images taken of huNOG-EXL skulls, **D,** Quantified bioluminescence of huNOG-EXL skull metastasis.

**Figure S4: Metastasis by 22Rv1 to the spleen in huNOG and huNOG-EXL mice. A,** Bioluminescent images taken of huNOG spleens, **B,** Quantified bioluminescence of huNOG spleen metastasis. **C,** Bioluminescent images taken of huNOG-EXL spleens, **D,** Quantified bioluminescence of huNOG-EXL spleen metastasis.

**Figure S5: Metastasis by 22Rv1 to the lung in huNOG and huNOG-EXL mice. A,** Bioluminescent images taken of huNOG lungs, **B,** Quantified bioluminescence of huNOG lung metastasis. **C,** Bioluminescent images taken of huNOG-EXL lungs, **D,** Quantified bioluminescence of huNOG-EXL lung metastasis.

**Figure S6: Metastasis by 22Rv1 to the heart in huNOG and huNOG-EXL mice. A,** Bioluminescent images taken of huNOG hearts, **B,** Quantified bioluminescence of huNOG heart metastasis. **C,** Bioluminescent images taken of huNOG-EXL hearts, **D,** Quantified bioluminescence of huNOG-EXL heart metastasis.

**Figure 7: Percentage of CD3+ cells showing TNF-α expression via intracellular staining isolated from subcutaneous 22Rv1 tumors huNOG mice.**

**Figure S8: 22Rv1 growth in huNOG-EXL and NOG mice. A,** Subcutaneous “primary” flank tumor volume growth measured over time in both huNOG-EXL and NOG-EXL mice. huNOG: Test n=7, Intact n=7, Castrated n=7, Enza n=8. NOG: Test n=4, Intact n=4, Castrated n=5, Enza n=3. **B,** Subcutaneous “primary” flank tumor volume growth measured over time in both huNOG-EXL huNOG: Test n=14, Intact n=14, Castrated n=14, Enza n=16. NOG: Test n=8, Intact n=8, Castrated n=10, Enza n=6. **C,** Subcutaneous “primary” flank tumor volume growth measured over time in both NOG-EXL.

**Figure S9: Immune-profile in huNOG-EXL 22Rv1 xenograft spleen. A,** Gating strategy used to determine presence of human CD45+ cells in the NOG-EXL model spleen. **B,** Representative data showing the abundance of various immune cell populations; human leukocytes, CD19+ cells, CD3+ cells and double negative cells, MDSC and activated myeloid cells and helper T-cells (CD4+) and cytotoxic T-cells (CD8+) (top to bottom). **C,** Quantitated data comparing human CD45, CD3, CD4, and CD11b populations in spleens isolated from testosterone implanted vehicle, intact vehicle, castrated vehicle, and castrated enzalutamide treated mice. **D,** Gating strategy for determining the activation state of MDSCs (CD3-CD19- CD11b+ CD14-). **E,** Representative data showing the activation state of the MDSC cells harvested from spleens under different treatment categories through the presence of the surface markers: CD25, CD44, CD69 and PD-1. **F,** Quantitated data showing the activation state of the spleen MDSCs throughout the different treatments.

**Figure S10: Immune-profile of T-cells in huNOG-EXL spleen. A,** Gating strategy for determining the activation state of T-cells and regulatory-like T-cells. **B,** Representative data showing the activation state of the CD3+ cells harvested from spleens under different treatment categories through the presence of the surface markers: CD25, CD44, CD69 and PD-1 and regulatory-like cells (CD3+CD25+PD-1+). **C,** Quantitation showing the expression of CD25, **D,** CD44, **E,** CD69, **F,** PD-1, **G,** regulatory-like T-cells (Tregs).

**Figure S11: Immune-profile of spleen and tumor B-cell Populations and B-cell activation NK-Cell population in huNOG-EXL spleen. A,** Representative data showing the activation state of the CD11b+CD14+ cells harvested from spleens under different treatment categories through the presence of the surface markers: CD25, CD44, CD69 and PD-1. **B,** Quantitation showing the expression of CD25, CD44, CD69, PD-1. **C,** Representative data showing the activation state of the CD19+ cells harvested from spleens under different treatment categories through the presence of the surface markers: CD25, CD44, CD69 and PD-1. **D,** Quantitation showing the expression of CD25, CD44, CD69, PD-1. **E,** Percent total CD19+ cells of the total CD45+ population of TILs. **F,** Percent total CD19+ cells of the total CD45+ population of splenocytes. **G,** Percent total NK cells from the spleen (CD3-CD19-CD16+CD56+).

**Figure S12: Multi-IF for CD3, CD8 and Granzyme B of 22Rv1 tumors in huNOG-EXL mice.** DAPI nuclear stain (top row), CD3 (green, second row), CD8 (red, third row), Granzyme B (purple, fourth row), and merged (last row) representative images of stained subcutaneous tumors from either castrated/testosterone implant (TEST, left column), intact/vehicle (INT, second column from left), castrated/vehicle (CAST, third column from left) or castrated/enzalutamide (ENZA, fourth column from left) mice, with a representative metastasis in the liver (from INT group, fifth column from left), and normal liver (sixth column from the left) and lymph node (rightmost column), as normal tissue control and positive staining controls, respectively.

**Figure S13: IHC of AR and PD1 of 22Rv1 tumors in huNOG-EXL mice.** H&E at 20x (top row), AR (middle row), and PD-1 (last row) staining from serial sections of 22Rv1 subcutaneous tumors from either castrated/testosterone implant (TEST, left column), intact/vehicle (INT, second column from left), castrated/vehicle (CAST, third column from left) or castrated/enzalutamide (ENZA, fourth column from left) mice, with a representative metastasis in the liver (from INT group, rightmost column).
